# Strategies for optimizing CITE-seq for human islets and other tissues

**DOI:** 10.3389/fimmu.2023.1107582

**Published:** 2023-03-01

**Authors:** Sarah J. Colpitts, Matthew A. Budd, Mahdis Monajemi, Kyle T. Reid, Julia M. Murphy, Sabine Ivison, C. Bruce Verchere, Megan K. Levings, Sarah Q. Crome

**Affiliations:** ^1^ Department of Immunology, Temerty Faculty of Medicine, University of Toronto, Toronto, ON, Canada; ^2^ Toronto General Hospital Research Institute, Ajmera Transplant Centre, University Health Network, Toronto, ON, Canada; ^3^ Department of Surgery, University of British Columbia, Vancouver, BC, Canada; ^4^ BC Children’s Hospital Research Institute, Vancouver, BC, Canada; ^5^ Department of Pathology and Laboratory Medicine, Canada and Centre for Molecular Medicine and Therapeutics, University of British Columbia, Vancouver, BC, Canada; ^6^ School of Biomedical Engineering, University of British Columbia, Vancouver, BC, Canada

**Keywords:** CITE-seq, tissue immunity, flow cytometry, pancreas, single cell RNA seq

## Abstract

Defining the immunological landscape of human tissue is an important area of research, but challenges include the impact of tissue disaggregation on cell phenotypes and the low abundance of immune cells in many tissues. Here, we describe methods to troubleshoot and standardize Cellular Indexing of Transcriptomes and Epitopes by sequencing (CITE-seq) for studies involving enzymatic digestion of human tissue. We tested epitope susceptibility of 92 antibodies commonly used to differentiate immune lineages and cell states on human peripheral blood mononuclear cells following treatment with an enzymatic digestion cocktail used to isolate islets. We observed CD4, CD8a, CD25, CD27, CD120b, CCR4, CCR6, and PD1 display significant sensitivity to enzymatic treatment, effects that often could not be overcome with alternate antibodies. Comparison of flow cytometry-based CITE-seq antibody titrations and sequencing data supports that for the majority of antibodies, flow cytometry accurately predicts optimal antibody concentrations for CITE-seq. Comparison by CITE-seq of immune cells in enzymatically digested islet tissue and donor-matched spleen not treated with enzymes revealed little digestion-induced epitope cleavage, suggesting increased sensitivity of CITE-seq and/or that the islet structure may protect resident immune cells from enzymes. Within islets, CITE-seq identified immune cells difficult to identify by transcriptional signatures alone, such as distinct tissue-resident T cell subsets, mast cells, and innate lymphoid cells (ILCs). Collectively this study identifies strategies for the rational design and testing of CITE-seq antibodies for single-cell studies of immune cells within islets and other tissues.

## 1 Introduction

Type 1 diabetes (T1D) is an autoimmune disease characterized by T-cell mediated destruction of insulin-producing beta cells in pancreatic islets (1). The balance between beta cell function and regeneration versus dysfunction and death is influenced by a variety of islet-proximal immune cells such as macrophages and other innate cells, as well as effector and regulatory T cells and other lymphoid populations (2–7). However, much of our current understanding comes from studies in mice (8–12) and there is a need to better understand cellular-cross talk mechanisms that control the function of human islets in both health and T1D. Characterization of human islet-resident immune cells and their interactions has proven challenging, however, due to low frequency of immune cells within islets and effects of dissociating tissue that can impact surface antigens (13–17).

Phenotyping of human tissue-resident immune cells has been significantly advanced by the advent of Cellular Indexing of Transcriptomes and Epitopes by sequencing (CITE-seq). This method allows simultaneous capture of cell surface protein and messenger RNA (mRNA) expression of single cells (18), and is particularly useful for detecting immune cell lineage markers with low mRNA expression (19) as well as unbiased capture of the transcriptome of novel cell types (20). For example, T cell populations such as γ/δ and mucosal-associated invariant T cells, and cell types such as innate lymphoid cells (ILCs) and neutrophils are not well identified by single-cell RNA sequencing due to low RNA content of lineage defining transcripts, high levels of RNase (20–22), and mRNA expression patterns that do not correlate with protein expression (19). Thus, annotating immune populations solely on the basis of mRNA expression can lead to misidentification or an inability to distinguish distinct populations with overlapping transcriptional characteristics.

Despite the advantages of assessing surface protein expression using CITE-seq, there are several methodological challenges. One obstacle is the identification of optimal antibody titrations, as hyper-concentration can lead to high background signal and increased sequencing costs without adding sequencing depth, whereas insufficient antibody can lead to insufficient signal to distinguish positive expression patterns (23). Flow cytometry is often used as a surrogate to define CITE-seq antibody titrations, on the basis of the assumption that the signals of oligo-tagged antibodies correlate to those from the same clone in a fluorochrome-tagged format (18). However, due to differences in antibody lots, tissue source, fluorescence spillover, tissue autofluorescence, and non-specific background binding, optimal concentrations of flow cytometry versus CITE-seq antibodies may differ.

Another challenge in studying human tissue samples is the use of enzymatic digestion to create single cell suspensions. The type of enzymes used and length of digestion time can significantly affect the presence of cell surface proteins (24). For islets, a variety of purified digestive enzymes can be used during the isolation process for clinical or research applications, including collagenase NB1 (Nordmark; Uetersen, Germany), Liberase™ (Roche; Basel, Switzerland) and/or Collagenase Gold (Vitacyte; Indianapolis IN, USA) (25, 26). The comparison between these collagenase enzymes used in islet isolation shows that they produce similar islet purity and viability (27). During the tissue digestion process, cell surface molecules on both immune and parenchymal populations may be damaged (17, 24), necessitating assessment of the digestion-induced destruction of epitopes of interest to accurately assess the phenotype of resident immune cells (17).

Herein, we assessed the impact of pancreas digestion and islet isolation on extracellular immune cell lineage and phenotype markers, and identified antibody clones that are sensitive or resistant to the digestion process. We also optimized titration of antibodies for CITE-seq using flow cytometry, and characterized expression of immune cell markers in healthy human islets by paired flow cytometry and CITE-seq.

## 2 Materials and methods

### 2.1 Experimental design

**Figure 1:**
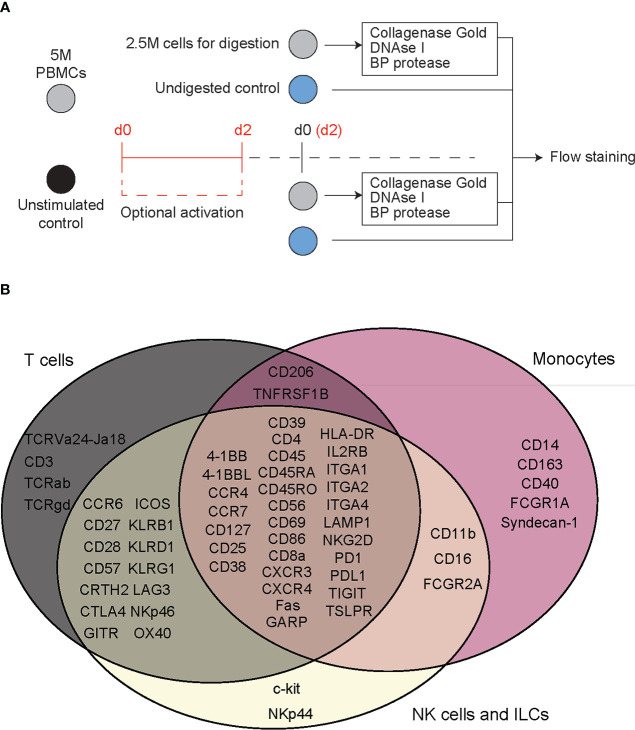
Flow cytometry reveals T cell specific, deleterious effects of islet digestive enzymes on staining of antibody clones used for generation of TotalSeq-C oligo-antibodies. **(A)** Activated or non-activated PBMCs were treated with a digestive enzyme solution and stained with flow cytometry antibodies corresponding to clones used in the TotalSeq-C commercial antibody catalogue of oligo-antibodies used for CITE-seq. **(B)** Markers were selected to identify key cellular subsets of T lymphocytes, monocytes, and innate lymphoid cells, in addition to other antibodies used for e.g., leukocyte selection, stress indicators, and immune activation.

To evaluate the effect of digestive enzymes used during islet isolation on CITE-seq oligo-antibodies, peripheral blood mononuclear cells (PBMCs) were treated with enzymes to mimic the process used by the University of Alberta IsletCore (26). Splenocytes were used in during CITE-seq antibody titrations. All donor information can be found in **Supplemental Table S1**. Cells were incubated for 30 minutes, with or without digestion enzymes, and then stained with a variety of antibody panels to comprehensively classify and characterize T cell-, myeloid-, and ILC-derived subpopulations. We then compared the proportion of cells positive for each antibody stain in a common parent cell type: either lymphoid or myeloid, depending on the marker of interest (**Figure 1A** and **Supplemental Figure S1**).

### 2.2 PBMC and spleen preparation

Human tissue specimens were collected in accordance with biosafety and ethical protocols approved by the University of British Columbia Clinical Research Ethics Board (B22-0075 and H18-02553, respectively) and Canadian Blood Services, and the University Health Networks Research Ethics Board and biosafety protocols (17-6229 and 20-5206, respectively). PBMCs were derived from venous blood and cryopreserved in aliquots as previously described (28).

For digestion experiments, PBMCs were thawed in a 37°C water bath and transferred to pre-warmed (37°C) X-VIVO cell culture media containing 5% human serum at a concentration of 1 million cells/mL. For panels consisting of markers requiring immune activation, cells were divided into two equal-volume aliquots, one of which was activated with CytoStim™ polyclonal T cell stimulant to a concentration of 1:200 (stimulant to media) and cultured for 48 hours at 37°C.

Islet perfusion solution was prepared following a standardized protocol from the University of Alberta (26) from HBSS with 3.6 mM calcium chloride, 0.81 mM magnesium sulfate, 4.2 mM sodium bicarbonate, 10 mM HEPES, and 100 U penicillin-streptomycin adjusted to a pH value of 7.35. Perfusion buffer was combined with a digestion solution of 2.8 mg/mL Collagenase Gold, 12,500 U/g BP Protease, and 5.6 mg/mL DNAse I, Grade II. Up to 2x10^6^ PBMCs were then incubated for 30 min at 37°C in the combination perfusion buffer/digestive solution. The vials were gently agitated at 10-minute intervals. After digestion cells were washed in PBS containing 0.5 mM EDTA and resuspended in 4.5 mL PBS/EDTA. Trypsin (390 µL of 0.25%) was added to the cell suspension and incubated at 37°C for 10 min. The reaction was stopped by adding 10 mL PBS containing 1% fetal bovine serum (**FBS**), 1 mM EDTA, and 11 mM GlutaMAX™. Finally, cells were transferred to a 96-well V-bottom polystyrene plate for flow cytometry staining.

Spleens were received from the University of Alberta IsletCore or the Ajmera Transplant Centre Islet Transplant Program. Samples were cut into small pieces using a sterile scalpel then placed into gentleMACS C-tubes with 10 mL PBS plus 2% FBS and placed in a gentleMACS dissociator using the m_spleen_01_01 setting. After dissociation, the slurry was mashed through a 70 µm cell strainer and ACK lysed to remove red blood cells (29). Cell pellets were resuspended in 50 mL RPMI media and 1x10^6^ cells were removed per well, centrifuged and resuspended in 50 µL master mixes containing cell staining buffer and either flow cytometry or CITE-seq antibodies.

### 2.3 Flow cytometry of PBMCs to assess clonal sensitivity to enzymatic digestion

**Figure 2:**
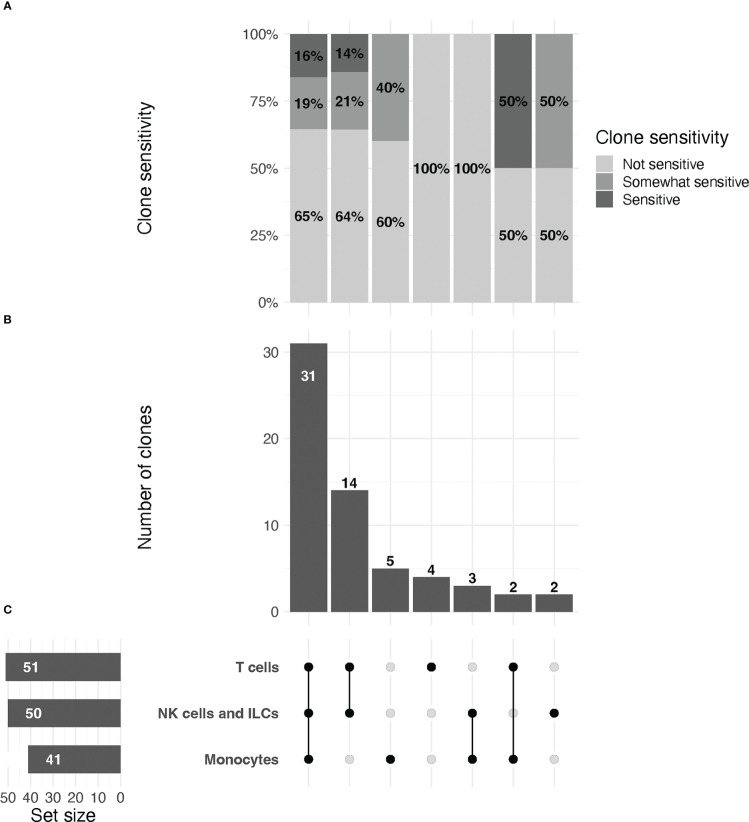
UpSet plot of clones included in the study, organized by immune cell lineage (T cell, NK/ILC, or monocyte).**(A)** Relative proportions of sensitive, partially sensitive, and insensitive clones per grouping. **(B)** Histogram representing number of clones tested per grouping. (**C**, left) From top to bottom, total number of clones tested for markers that are expressed by T cells, NK/ILCs, and monocytes, respectively. Line plot (right) signifies groupings of markers for data in **(A, B)** that are expressed by one, two, or all three cell types, as indicated by connected dots (e.g., the first column describes data for markers that are expressed by all three cell types, the second column for markers expressed only by T cells and NK/ILCs, etc.). Groupings are exclusive.

PBMCs, either exposed to islet perfusion solution or unexposed controls, were stained with a combination of the 61 anti-human antibodies listed in **Supplemental Table S2**. Each staining panel was selected to distinguish key subpopulations of myeloid cells and lymphocytes. For each cell surface marker, at least one of the antibody clones tested matched that of the corresponding marker in BioLegend’s TotalSeq™-C Human Universal Cocktail selection kit. Panels were designed to allow standard lineage gating of immune cell phenotypes whenever possible. Samples for flow cytometry were acquired on the FACS Symphony A5 platform and analyzed using FlowJo software (BD Biosciences; version 10.8.1). All events were gated on live, single lymphoid or myeloid populations as applicable (gating strategy shown in **Supplemental Figure S1**). Gates were set on undigested cells and then applied to the digested cells. Markers were assigned to either a myeloid lineage or activated/non-activated lymphocyte lineage, and percent positive of parent populations reported. Figures were generated with Prism 9 (GraphPad Software; version 9.3.1) and the R statistical computing environment (version 4.2.0). **Figure 2** was generated using the ComplexUpset package (30).

All data pertaining to fluorophore-conjugated antibody staining of cells treated with digestion enzymes were considered for quality-related inclusion or exclusion from the study at the time of data collection and analysis. Our *a priori* criteria for inclusion of data in the final assessment were as follows: 1) Data for n=2 or more PBMC donors; 2) a population of positive cells clearly identifiable in the undigested cell sample; 3) for markers on activated cells, a sufficient activation signal, determined *via* CD69 staining, must be visible in the flow cytometry output.

Readings of identical clone-fluorophore combinations during a single flow cytometry experiment (e.g., repeat measurements from different staining panels on the same day) were used to determine mean values and recorded in the final dataset. Digestion sensitivity of technical replicates (or the mean value for repeated measurements) for each antibody was assessed by calculating the relative change (**RC**) of the positive population in digested cells (to undigested cells) and converting to a percentage (see **Equation 1**).


$$RC=100*\frac{\%\;positive\;\left(undigested\right)-\%\;positive\;\left(digested\right)}{\%\;positive\;\left(undigested\right)}$$

**Equation 1**. Expression for quantifying relative change in positive staining for flow cytometry antibodies in digested and undigested cells.

A sample was considered sensitive if the RC was ≥ 50, and partially sensitive if 25 ≤ RC ≤ 50. As the equation is biased to output large values for small inputs (e.g., markers for rare cell populations), replicates with ≤ 5% positivity of the undigested cells were assessed for sensitivity *via* standard flow gating. If a positive population for the antibody was visible, it was considered insensitive to digestion.

Subsequently, overall clone sensitivity to digestion was assessed by computing the proportion of replicates for each clone with full or partial sensitivity. Antibody clones were considered sensitive if the majority of replicates had an RC ≥ 50; partially sensitive if the majority of clones had an RC ≥ 25 but ≤ 50; and insensitive if the majority of replicates had an RC ≤ 25. Clones with a 50% split between full/partial or partial/insensitive replicates were categorized as partially sensitive.

### 2.4 Human islet preparation

Human islets were received in accordance with research ethics protocols 20-5206 (UHN) and H20-01930 (UBC). Human islets (~10,000 islet equivalents) were obtained from the IsletCore (University of Alberta) and shipped overnight in CMRL 1066 media. Prior to CITE-seq staining, islets were dissociated into a single cell suspension by centrifugation (800 rpm, 5 min), resuspended in 5 mL trypLE, and incubated in a 37°C water bath. After 2 minutes, the islets were removed, pipetted vigorously, and returned to the water bath for an additional 3 minutes. Islets were counted and immune cells enriched using an Easysep human CD45 Depletion kit II. Cells were resuspended at 1.0x10^8^ cells/mL in EasySep™ buffer and 12.5 µL/mL EasySep Human CD45 Depletion Cocktail II was added and incubated for 5 minutes at room temperature. EasySep Dextran RapidSpheres (20 µL/mL) were then added and incubated for 3 minutes at room temperature. The mixture was then topped up to 2.5 mL with EasySep buffer and placed in an EasySep purple magnet for 5 minutes. The CD45-negative fraction was decanted, and the positive fraction added to the magnet for an additional 5 minutes in 2.5 mL EasySep buffer. The resulting CD45-positive fraction was then counted, 1x10^6^ cells were removed, centrifuged, and resuspended in 50 µL master mix containing cell staining buffer and flow cytometry antibodies or TotalSeq-C antibodies for CITE-seq, or a mix of both for ILC enrichment.

As ILCs are present in very low abundance and display significant overlap in protein and RNA level expression of molecules expressed by T cells, we also performed an ILC enrichment on the same sample. CD45-enriched islet cells (1x10^6^) were stained with flow cytometry antibodies as well as the TotalSeq™-C antibody cocktail (at a 1:1 ratio) and flow sorted before sequencing. FITC-conjugated antibodies against B cells, T cells, and myeloid cells were used to differentiate these immune cells from ILCs. Antibodies used for lineage exclusion are listed in **Table 1**. We also used a live/dead dye (FVS700) and antibodies directed against CD45 (APC Cy7, clone HI30), CD56 (BV605, clone HCD56), and CD127 (PE, clone hIL-7R-M21) to differentiate helper ILCs from NK cells. Cells were sorted as Live, CD45^+^, Lineage^-^ (**Supplemental Figure S2**) washed as above and sent for sequencing.

**Table 1:** Flow cytometry antibodies used to identify cells positive for classical immune cell lineage markers.

Antibody	Clone
CD3	OKT3
CD3	UCHT1
CD4	RPA-T4
CD8a	RPA-T8
CD14	M5E2
CD15	W6D3
CD19	HIB19
CD20	2H7
TCR a/b	IP26
TCR g/d	B1
CD33	HIM3-4
CD34	583
CD203c	NP4D6
FceR1a	AER37
CD79a	HM47
CD138	MI15

### 2.5 Assessing optimal antibody titrations using splenic samples

To capture tissue-resident myeloid populations, such as those seen in islets, we used human splenocytes for flow cytometry-based CITE-seq antibody titrations. Splenocytes were thawed in pre-warmed, serum-free RPMI, washed and resuspended at 5x10^6^ cells/mL in RPMI containing 5% human serum and 1% penicillin/streptomycin. Since some markers of interest were only expressed upon activation, cells were either activated (with LPS + IFNγ or PMA/ionomycin) or rested for 6 hours in complete RPMI at 37°C. If the marker of interest was expressed more abundantly on myeloid lineage cells, cells were activated with 10 ng/mL LPS + 100 ng/mL IFNγ. If the marker of interest was expressed more abundantly on ILCs or T cells, cells were activated in PMA/Ionomycin cell stimulation cocktail. After stimulation, 3x10^6^ cells were washed with PBS plus 2% FBS (FACS buffer), resuspended in 75 µL Human TruStain FcX™ Fc Blocking reagent (at a 1:10 dilution in FACS buffer) and 1x10^6^ cells (25 µl of Fc blocked cells) were plated in a V-bottom plate for 15 minutes at 4°C. Each antibody was tested in 3 dilutions: 2x, 1x and 0.5x the recommended dilution and added to 25µl of FACS buffer. Samples were stained for 30 minutes at 4°C and washed in FACS buffer. Acquisition was performed on a BD LSRFortessa flow cytometer and analyzed using FlowJo software (BD Biosciences; version 10.8.1).

### 2.6 CITE-seq staining of spleen and islets

Surface staining was performed as described in the BioLegend protocol (31). Briefly, 1x10^6^ cells were resuspended in 45 μL Cell Staining Buffer in 1.5 mL microcentrifuge tubes. Human TruStain FcX™ Fc Blocking reagent (5 µL) was added, and cells were incubated for 10 minutes at 4°C. TotalSeq™-C antibody cocktails were made during the incubation using concentrations determined by previous flow-cytometry-based CITE-seq titrations. All TotalSeq™-C antibodies added can be found in **Supplemental Table S3**. The resulting cocktail was then added to cells and incubated for 30 minutes at 4°C. After incubation, cell pellets were resuspended with 1 mL PBS plus 0.05% BSA and centrifuged for 5 minutes at 400*g. The wash was repeated twice more for a total of 3 washes, and the final concentration was adjusted to 1200 cells/µL and sent for sequencing to a local biomedical research core.

### 2.7 CITE-seq data analysis pipeline

Samples were prepared for sequencing using the 10X Genomics Single Cell 5’ v2 platform in accordance with manufacturer’s instructions for capture of 12,000 cells per sample. Reverse transcription, cDNA amplification and sequencing libraries were generated using 10X Genomics Single Cell 5’ v2 reagents. Across samples, cells were sequenced to a target depth of 40,000 reads per cell. Read alignment to the reference human genome (GRCh38/hg38) and gene expression matrices were generated by the 10X Genomics CellRanger pipeline (version 6.1.2) 7. In line 640 please remove “to generate UMAPS.

Data were loaded into R and Seurat objects were created individually for both islets and spleen. High mitochondrial content cells were removed from the islet clusters by removing cells with >10% of Unique Molecular Identifiers (**UMIs**) mapped to mitochondrial genes and <200 unique genes. For splenocytes, cells which had >20% of UMIs mapped to mitochondrial genes and <200 unique genes were removed. Data were normalized with SCTransform (32), principal component analysis was used for dimensionality reduction (RunPCA) and cells were clustered using the Louvain algorithm with 30 principal components (FindNeighbors and FindClusters) in Seurat (33). Clusters were visualized using the Uniform Manifold Approximation and Projection (**UMAP**) algorithm (34).

In each object, immune cells (identified as clusters expressing *PTPRC*), were used to create an immune cell-only object. The individual datasets were then merged and integrated using harmony (RunHarmony) (35). Cell type-specific thresholds were set to remove low quality cells and all cells with >10% of UMIs mapped to mitochondrial genes, as above. Cells with low transcript abundance (<200 features) and high antibody expression indicative of antibody aggregates were removed. Integrated data was then normalized following the same process above. Cell types and lineages were annotated by analysing the top differentially expressed genes and/or proteins (FindMarkers) and manually labelled. T cell UMAPs were generated by selecting cells which expressed either *CD3E* or CD3 and re-clustered.

## 3 Results

### 3.1 Epitopes for several antibody clones in the TotalSeq™-C kit are affected by digestive enzymes

We performed flow cytometry on PBMCs which were or were not exposed to a mock islet digestion protocol using a total of 92 antibody clones specific for 64 immune cell markers (**Figure 1**). Surface marker expression by cell type is displayed in **Figure 1B**. Up to five antibody clones were tested per marker, with 2-18 replicates per clone on cells from 2-4 PBMC donors. In the final analysis, data from 75/92 (82%) of tested clones, specific for 61 immune cell markers were included.

To assess epitope sensitivity to digestive enzymes, we first analyzed data *via* traditional flow cytometry to identify potentially problematic clones/markers for which no positive population was observed in the digestion condition. We then assessed the quantity of cells positive for each marker as a proportion of the parent cell population.

Across all replicates included in the final analysis (n=216), we observed a median relative change of 8.2 (IQR: -1.1 to 52.7), with values ranging from –188% to 100% (Note: RC values correspond to relative change of mean fluorescence intensity for the marker of interest in digested versus undigested cells). We found that 37/216 (17%) replicates displayed low, but quantifiable, relative expression in the parent population of cells in both the digested and undigested samples, and we treated these replicates for reporting purposes as non-sensitive. A total of 116/216 (54%) replicates were non-sensitive with >5% positivity in the undigested parent cells (i.e. true non-sensitives), 20/216 (9%) partially sensitive, and 43/216 (20%) significantly sensitive. A summary of these observations stratified by immune cell type is provided in **Figure 2**.

**Figure 3:**
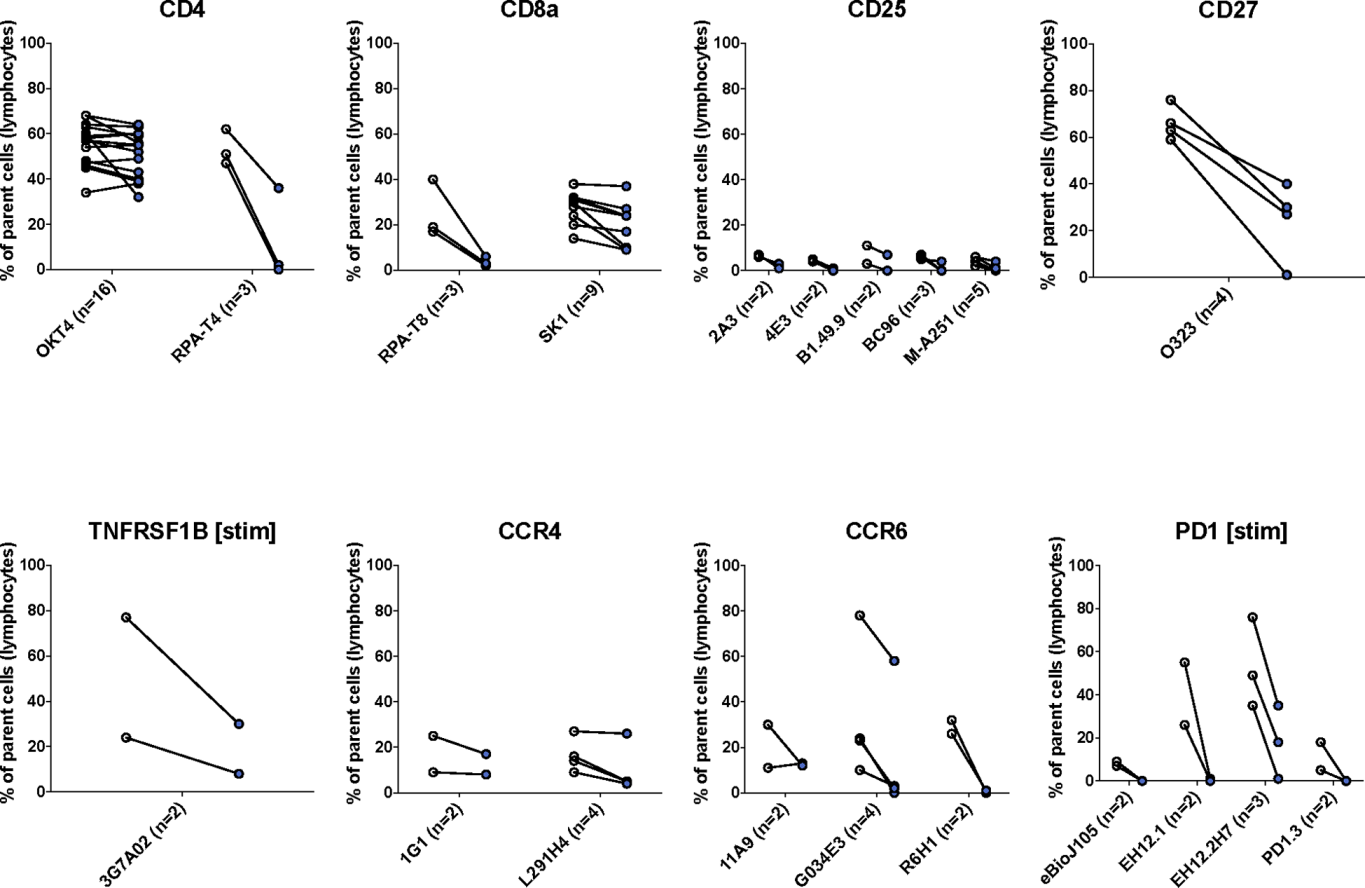
The effects of enzymatic digestion on key phenotypic markers of concern in the TotalSeq-C antibody library. White and blue circles indicate pre- and post-digestion values, respectively. Each pairing signifies an independent experimental replicate of pre- and post-treatment measurements, and the proportion of cells positive for the marker of interest was determined using standard flow gating. Parent cells were total live lymphocytes after applying quality control gating as outlined in **Supplemental Figure S1**.

**Figure 4:**
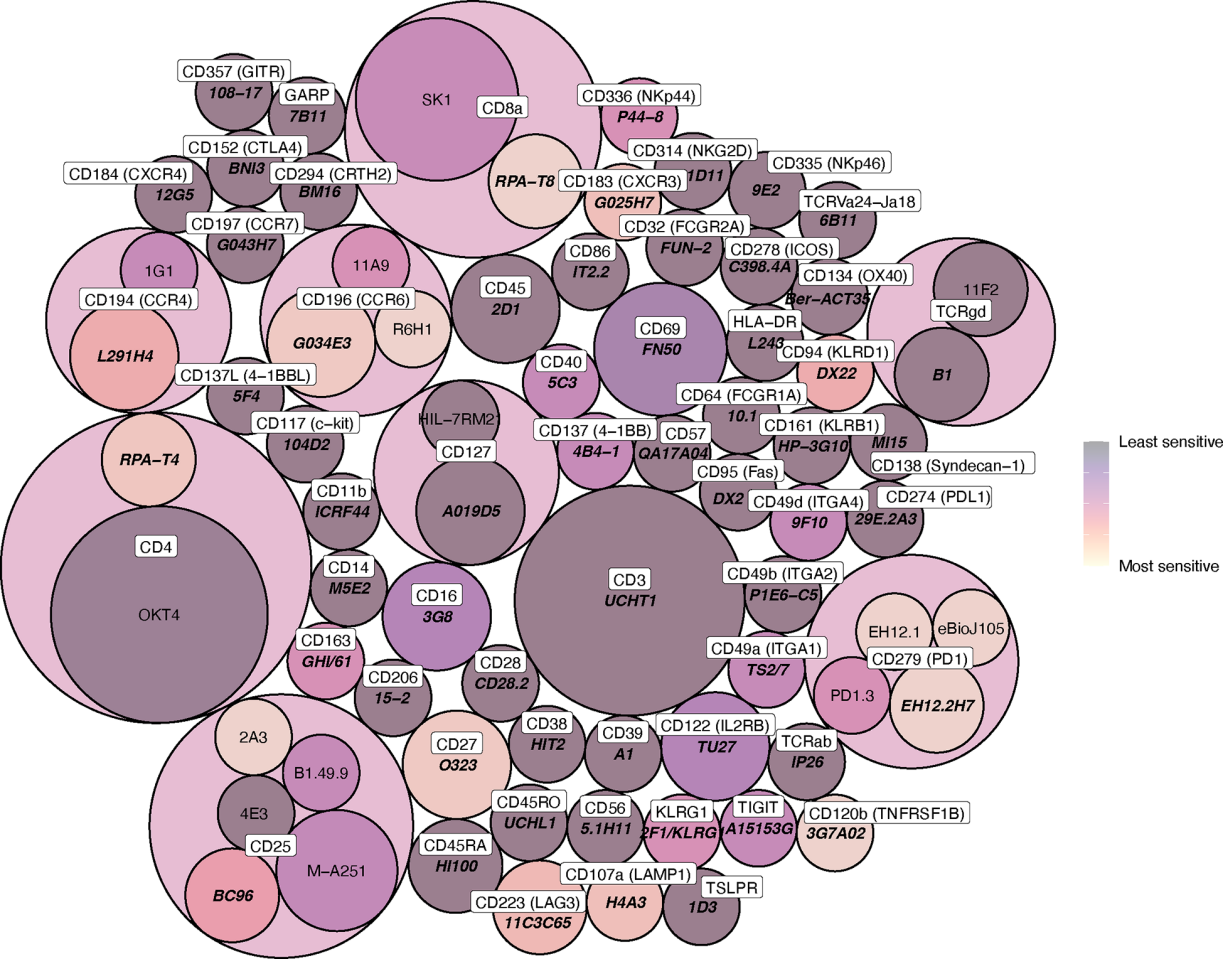
Circle packing heat map of clone sensitivity to digestive enzymes. The colour scale indicates relative proportion of replicates sensitive or partially sensitive to digestion, and the size of each circle indicates the number of replicates included in analysis. Circle containers indicate successive levels of hierarchy, where clones are grouped according to immune marker (the colour of these encapsulating hierarchy circles, which contain multiple daughter clones, is not indicative of daughter clone sensitivity).

We found that most (47/75, 63%) antibody clones tested using flow cytometry that align with TotalSeq-C oligo-antibodies were not sensitive to the enzymatic digestion process, with some notable exceptions (**Figure 3**; **Table 2**). We observed partial sensitivity in n=16 (21%) of clones tested, and high sensitivity in n=12 (16%) of clones. A library of figures for each marker is included as a supplementary download, indicating the proportion of parent cells positive for each antibody stain and subsetted by clone tested. Of the n=12 clones found to be highly sensitive, n=8 (75%) belonged to the TotalSeq-C library, summarized in **Table 2**. Additionally, **Figure 4** summarizes the sensitivity of each clone included in our final results in a hierarchical heatmap format.

**Table 2:** List of clones tested for key markers of concern.

Clone tested	Clone with best observed staining	Number of donors> clone was tested with	Number of replicates included	Proportion of replicates sensitive (%)	Proportion of replicates partially sensitive (%)	Proportion of replicates not sensitive (%)
CD4	OKT4					
* OKT4*		4	16	0	0	100
***RPA-T4***		2	3	67	33	0
CD8a	SK1					
***RPA-T8***		2	3	100	0	0
* SK1*		3	9	22	22	56
CD25 (IL2R)	4E3					
* 2A3*		2	2	100	0	0
* 4E3*		2	2	0	0	100
* B1.49.9*		2	2	0	50	50
***BC96***		3	3	67	0	33
* M-A251*		3	5	20	20	60
CD27	O323					
***O323***		2	4	75	25	0
CD120b (TNFRSF1B)	3G7A02					
***3G7A02***		2	2	100	0	0
CD194 (CCR4)	1G1					
* 1G1*		2	2	0	50	50
***L291H4***		3	4	75	0	25
CD196 (CCR6)	11A9					
* 11A9*		2	2	50	0	50
***G034E3***		3	4	75	25	0
* R6H1*		2	2	100	0	0
CD279 (PD1)	PD1.3					
* eBioJ105*		2	2	100	0	0
* EH12.1*		2	2	100	0	0
***EH12.2H7***		3	3	100	0	0
* PD1.3*		2	2	50	0	50

TotalSeq-C clones highlighted in bold text.

### 3.2 Flow cytometry antibody titrations allow estimates of antibody concentrations for CITE-seq studies

**Figure 5:**
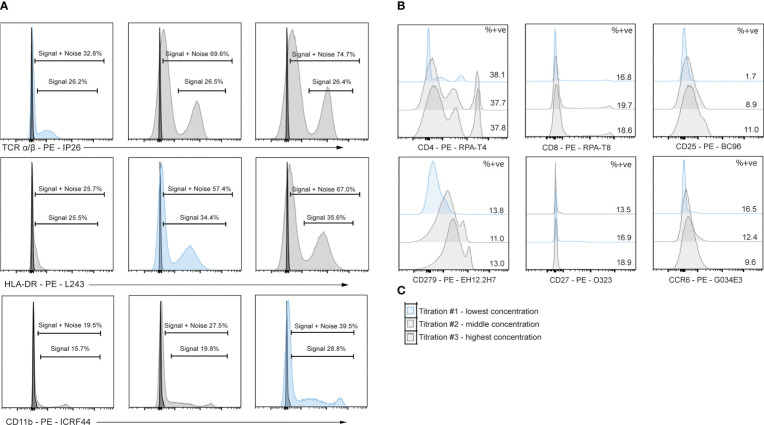
Titrations of flow cytometry antibodies corresponding to oligo-antibody clones in the TotalSeq-C catalogue reveals optimal staining conditions. PBMCs were stained using PE antibodies against the marker of interest and performed in 3-fold serial dilutions. Dilution chosen is shown in blue. **(A)** Example titrations showing percent positive noise + signal and percent positive signals. The titration chosen is the dilution that does not lose positive signal yet has the least signal + noise percent positive. **(B)** 3-point titrations performed on markers affected by enzymatic digestion. **(C)** Example legend for histograms shown in B, where the top plot is titration #1 and the lowest dilution, middle plot is titration #2 and the middle concentration, and the bottom plot is titration #3 and highest concentration of antibody.

Flow-based antibody titrations were used to determine the optimal concentration for CITE-seq staining, as prior studies suggested flow-based signal would be analogous to CITE-seq signals (18). PE-conjugated antibodies corresponding to the same markers and epitopes (clones) as the TotalSeq-C antibodies were utilized. Three titrations were performed on each antibody of interest with the middle concentration being that recommended by the vendor (BioLegend). Flow cytometric analysis was used to determine the lowest amount of PE-conjugated antibody needed to generate a positive signal (**Table 3**; **Figure 5**). As some populations of interest are rare or absent in PBMCs, we used human splenocytes to perform the antibody titrations. For markers which were more highly expressed upon activation, cells were activated for 6 hours before staining. For activation markers on myeloid cells (e.g., CD80, CD86 and CD163), cells were stimulated with a combination of LPS and IFNγ, and for those on T cells and/or ILCs (e.g. CD69, ICOS, and CD107a), cells were activated with PMA/Ionomycin. Concentrations were assessed by the ratio of percent positive signal plus noise (gated based on unstained) to the percent positive signal (gated based on peak separation). The optimal antibody concentration was determined to be that which gave a positive peak with the lowest positive signal plus noise (**Figure 5A**). Titration results for each marker are listed in **Table 3** and **Figure 5B** gives examples of titration plots for markers affected by enzymatic digestion and **Figure 5C** is the legend for these plots, with the titration concentration selected to move forward with shown in blue. For 15 antibodies, the recommended dilution (titration concentration #2) was appropriate. However, for only 1 marker (CD11b), the highest concentration (titration concentration #3) gave a positive signal without substantial background staining (signal – (signal + noise) ratio). For the remainder and majority of antibodies (47), we noted the lowest concentration, titration #1, to be optimal. We therefore moved forward with the appropriate concentration based on these results for CITE-seq studies.

**Table 3:** List of antibodies titrated, stimulation condition, recommended titration from the vendor and 3 point titration values.

Antibody	Stimulation condition	Recommended Titration	Titration 1	Titration 2	Titration 3
CD56	–	0.05 - 0.8	**0.05**	0.425	0.8
CD161	–	0.125 - 2	**0.125**	1.0625	2
CD117 (c-kit)	–	0.25-1	**0.25**	0.625	1
CD16	–	0.025 - 0.4	**0.025**	0.2125	0.4
TIGIT (VSTM3)	–	0.125 - 2	**0.125**	1.0625	2
CD335 (NKp46)	–	0.05 - 0.8	**0.05**	0.425	0.8
CD294 (CRTH2)	–	>0.5	0.5	**1.25**	2
CD127 (IL-7Rα)	–	0.05 - 0.8	**0.05**	0.425	0.8
CD196 (CCR6)	–	0.0125 - 0.2	**0.0125**	0.106	0.2
CD314 (NKG2D)	PMA	0.0625 - 1	0.0625	**0.53125**	1
CD336 (NKp44)	–	>0.5	0.5	**1.25**	2
CD94	–	0.025 - 0.4	**0.025**	0.2125	0.4
KLRG1 (MAFA)	–	0.1-0.5	**0.1**	0.3	0.5
CD183 (CXCR3)	–	0.05 - 0.8	**0.05**	0.425	0.8
TCR γ/δ	–	N/A	0.05	**0.425**	0.8
CD45	–	0.01-0.1	**0.01**	0.055	0.1
TCR Vα24-Jα18 (iNKT cell)	–	N/A	**0.5**	1.25	2
TCR α/β	–	0.015 - 0.24	**0.015**	0.1275	0.24
CD8a	–	N/A	**0.025**	0.2125	0.4
CD3	–	0.0125 - 0.2	**0.0125**	0.106	0.2
CD4	–	0.025 - 0.4	**0.025**	0.2125	0.4
CD138 (Syndecan-1)	–	N/A	0.05	**0.425**	0.8
CD14	–	0.025 - 0.4	**0.025**	0.2125	0.4
CD206 (MMR)	–	0.25-1	**0.25**	0.625	1
HLA-DR	–	0.0125 - 0.2	0.0125	**0.106**	0.2
CD45RA	–	0.03125 - 0.5	**0.03125**	0.2656	0.5
CD45RO	–	0.125 - 2	**0.125**	1.0625	2
CD25	PMA	0.025 - 0.4	**0.025**	0.2125	0.4
CD223 (LAG-3)	PMA	0.125 - 2	**0.125**	1.0625	2
CX3CR1	–	0.0625-1	**0.0625**	0.53125	1
TSLPR (TSLP-R)	–	>0.5	**0.5**	1.25	2
CD49b	–	0.025 - 0.4	**0.025**	0.2125	0.4
CD38	PMA	0.05 - 0.8	**0.05**	0.425	0.8
CD57 Recombinant	–	0.025 - 0.4	**0.0.25**	0.2125	0.4
CD49a	–	0.025 - 0.4	**0.025**	0.2125	0.4
CD278 (ICOS)	PMA	0.0625 - 1	**0.0625**	0.53125	1
CD357 (GITR)	PMA	N/A	**0.5**	1.25	2
CD39	PMA	0.0125 - 0.2	**0.0125**	0.10625	0.2
CD69	PMA	0.025 - 0.4	**0.025**	0.2125	0.4
CD279 (PD-1)	PMA	0.125 - 2	**0.125**	1.0625	2
CD152 (CTLA-4)	PMA	0.25-1	**0.25**	0.625	1
CD107a (LAMP-1)	PMA	0.0625 - 1	**0.0625**	0.53125	1
CD95 (Fas)	PMA	0.125 - 2	**0.125**	1.0625	2
CD134 (OX40)	PMA	0.125 - 2	**0.125**	1.0625	2
CD137L (4-1BB Ligand)	PMA	>0.5	**0.5**	1.25	2
CD40	LPS/IFN	0.025 - 0.4	**0.025**	0.2125	0.4
CD137 (4-1BB)	PMA	0.125 - 2	0.125	**1.0625**	2
CD194 (CCR4)	–	0.0125-0.2	0.0125	**0.10625**	0.2
CD27	–	0.005 - 0.08	0.005	**0.0425**	0.08
CD28	–	0.03125 - 0.5	**0.03125**	0.2656	0.5
GARP (LRRC32)	–	0.125-0.5	**0.125**	0.375	0.5
CD122 (IL-2Rβ)	–	0.0625 - 1	**0.0625**	0.53125	1
CD184 (CXCR4)	–	>0.5	**0.5**	1.25	2
CD49d	PMA	0.0125 - 0.2	**0.0125**	0.10625	0.2
CD274 (B7-H1, PD-L1)	PMA	0.0625-1	**0.0625**	0.53125	1
CD120b	LPS/IFN	N/A	0.01	**0.255**	0.5
CD80	LPS/IFN	0.25-1	0.25	**0.625**	1
CD32/Fcg RII	LPS/IFN	0.0125 - 0.2	0.0125	**0.10625**	0.2
CD11b	LPS/IFN	0.0125 - 0.2	0.0125	0.10625	**0.2**
CD64 (FCGR1A)	LPS/IFN	0.0125 - 0.2	0.0125	**0.10625**	0.2
CD86	LPS/IFN	0.005 - 0.08	0.005	**0.0425**	0.08
CD163	LPS/IFN	0.0625 - 1	0.0625	**0.53125**	1
CD197 (CCR7)	–	>0.5	**0.5**	1.25	2

All titration values are μg per 100μl with 1 million splenocytes. Chosen titration values are bolded. N/A stands for Not Available.

### 3.3 CITE-seq antibody concentrations determined by flow cytometry allow identification of islet-resident immune populations

To test the effects of enzymatic digestion on CITE-seq samples, enzymatically dissociated islets and donor-matched spleen (mechanically dissociated only) from one individual were stained with a panel of 67 oligo-tagged antibodies associated with myeloid, T cell and ILC populations (**Supplemental Table S3**) and sequenced. As we were primarily focused on how enzymatic digestion impacted epitopes on immune cells and how CITE-seq could aid in better delineation of immune cell subsets, the panel of antibodies selected included markers commonly used to distinguish immune cell subsets and activation states.

**Figure 6:**
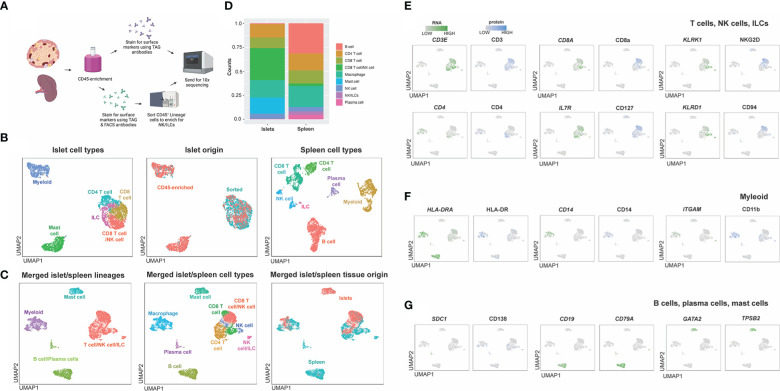
Workflow and combined islet and spleen lineage maps. **(A)** Example workflow of CITE-seq. Spleen and islet samples were enriched for CD45^+^ immune cells. Immune cells from spleen were stained with oligo-antibody conjugated TotalSeqC antibodies and sent for 10x sequencing. Islet immune cells were either were stained with oligo-antibody conjugated TotalSeqC antibodies or stained for both oligo-antibody conjugated TotalSeqC and flow cytometry antibodies at the same time and FACS sorted to enrich for NK cells and ILCs and then sent for 10x sequencing. **(B)** UMAPs of islet cell types, islet cell origin (FACS sorted or CD45-enriched), and spleen cell types. **(C)** Merged islet and spleen lineage, cell types and tissue of origin UMAPs. **(D)** Composition of cell types from each organ. **(E)** T, NK and ILC lineage markers. **(F)** Myeloid lineage markers. **(G)** B cell, plasma cell and mast cell lineage markers.

To ensure sufficient immune cells were captured by sequencing, CD45-enrichment using magnetic separation was performed on islets, as immune cells account for only 1-2% of cells within human islets (15). To further enrich for rare ILC populations, which can have overlapping transcriptomic signatures with CD4^+^ T cell subsets, ILCs were isolated *via* flow cytometry sorting by negatively gating on expression of lineage markers (**Table 1**) on islet-resident CD45^+^ cells (**Supplemental Figure S2**). This ILC-enriched sample was sequenced along with the donor-matched CD45-enriched sample (**Figure 6A**). Donor-matched spleen was also sequenced to serve as a control for assessing epitopes negatively impacted by enzymatic digestion and to aid in annotation of immune populations.

After sequencing, islet CD45-enriched and islet ILC-enriched samples were integrated (**Figure 6B**) and populations defined by RNA expression and antibody-derived tags (ADT) were compared to those observed in donor-matched spleen (**Figure 6B**). Individual islet and spleen UMAPs with clustering based on combined RNA expression and ADT were annotated based on cell types and origin (CD45-enriched or sorted) (**Figure 6B**). Next, islet-resident immune cells were merged with the splenocytes and normalization, PCA and cell-clustering analysis performed. Clusters in the merged islet and spleen dataset were then manually annotated based on cell lineage and cell type and compared across tissue of origin (islet or spleen) (**Figure 6C**). We noted the contribution and proportion of each cell type from islets or spleen differed (**Figure 6D**). Mast cells originated primarily from islets whereas B cells and plasma cells were predominantly from the spleen (**Figure 6D**). To annotate subsets of immune cell lineages, the expression of genes and proteins associated with T cells and ILCs (**Figure 6E**), myeloid cells (**Figure 6F**) and plasma cells, B cells and mast cells (**Figure 6G**) were assessed. Unique to islets, we also identified a CD8 T cell population with surface protein expression of NKG2D and CD94 (**Figure 6E**).

We noted, however, that although flow cytometric titrations were performed to optimize antibody dilutions for CITE-seq, the concentrations used based on this optimization were not always ideal, with some markers displaying a large degree of non-specific background signal. In particular, 4-1BBL, CD138, TCR γ/δ, CD64, NKp44 and OX40 contained notable background contamination. To correct this, minimum cut-offs were used to eliminate background signal from non-specific binding of oligo-tagged antibodies (**Supplemental Figure S3**).

**Figure 7:**
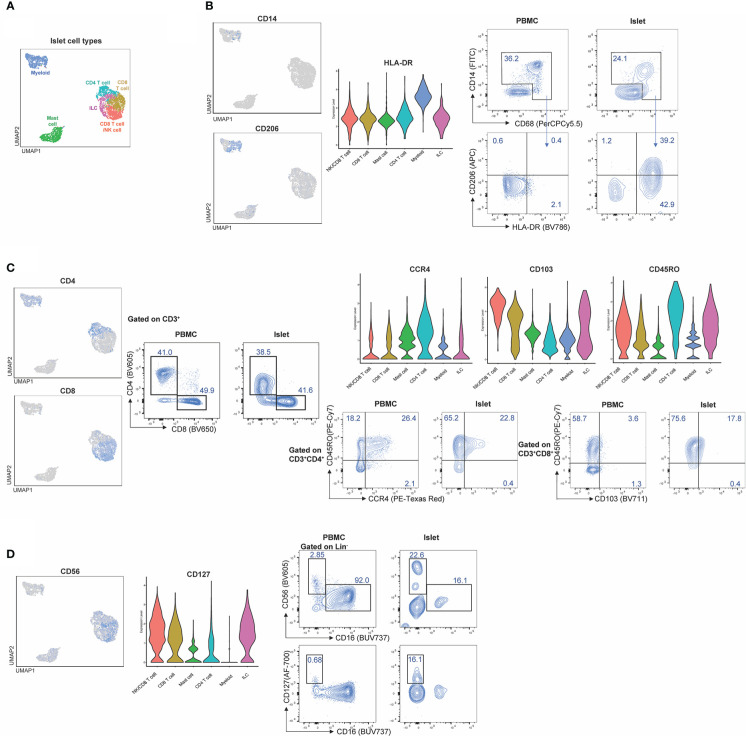
CITE-Seq protein expression is validated by flow cytometry on healthy human islets. Healthy human islets were analyzed by CITE-seq and flow cytometry. Surface marker proteins found to be expressed by islet-resident immune cells by CITE-seq were analyzed by flow cytometry in order to validate CITE-seq findings. PBMCs were used a staining control for flow cytometry analysis. **(A)** human islet UMAP, **(B)** Myeloid population analysis of CD14, CD206 and HLA-DR expression. **(C)** T cell population analysis of CD4, CD8, CD45Ro, CCR4 and CD103. **(D)** ILC analysis of CD56 and CD127.

To validate the CITE-seq protein data based on ADT, flow cytometry analysis was performed on islets from n=4 donors. Proportions of immune cells in healthy human islets (**Figure 7A**) were used to compare expression patterns in myeloid, T cell and ILC populations (**Figures 7B–D**). CD14 and CD68 were used to identify myeloid populations (**Figure 7B**) and the expression of HLA-DR and CD206 was validated in healthy human islets in comparison to PBMC control myeloid cells. CD3, CD4, and CD8 were used to identify T cell populations. CITE-seq revealed that islet-resident CD4^+^ T cells expressed CCR4 and CD45RO, which was also observed *via* flow cytometry (**Figure 7C**). In CD8^+^ T cells, high expression of CD103 and CD45RO was observed *via* both CITE-seq and flow cytometry (**Figure 7C**). Thus, there was concordance in positive expression between protein detected by flow cytometry and protein detected by CITE-seq in islet resident immune cells.

To examine whether combined RNA and protein data allowed for better delineation of cell types than either individually, we performed normalization and UMAP projections based on either RNA or ADT data alone from the merged islet and spleen dataset (**Supplemental Figure S4**). Manual annotation of the UMAPs was informed by heatmaps of select differentially expressed genes (DEGs) or differentially expressed proteins (DEPs) present in clusters defined by RNA, ADT, and/or combined RNA and ADT (**Supplemental Figure S5**). We found that analysis using RNA, ADT or RNA with ADT all generated UMAPs with 14 clusters (**Supplemental Figure S4A**). Populations labelled as ‘Unclear’ in **Supplemental Figure S4B** were those which lacked lineage defining markers by either RNA or ADT. Notably, manual annotation with information from either RNA or ADT alone did not match annotations derived from RNA and ADT (**Supplemental Figure S4B**). Specifically, clusters 0, 1, 2, 3, 4, 5, 8, 9, 11, and 14 were not accurately annotated using RNA or ADT data alone. Further, UMAPs derived from combined RNA and protein data were necessary to identify CD4^+^ T cells, NK/CD8^+^ T cells, NK cell/ILCs and CD8^+^ T cells as key lineage markers were not highly expressed at the RNA level. These findings emphasize the importance of including protein data to accurately distinguish T cell, ILC and NK cell types.

**Figure 8:**
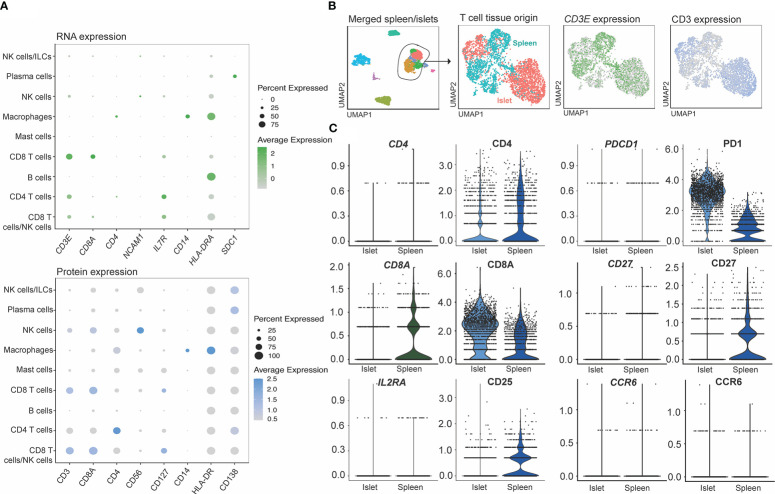
CITE-seq allows identification cell types through the expression of surface markers not captured by single cell RNA sequencing. **(A)** RNA (genes shown in green) and protein (surface markers shown in blue) expression of matched molecules. **(B)** Markers shown to be sensitive to digestion by flow cytometry on T cells. T cells subsets were extracted from the combined islet/spleen objects and **(C)** titration markers were compared between the digested (islets) and non-digested (spleen) samples.

We also assessed how protein and mRNA expression levels were correlated in human islet-resident immune cells by comparing lineage markers associated with T cells, ILCs, myeloid and plasma cells, as well as molecules associated with activation or inhibition (**Figure 8A**). Of the markers included in our CITE-seq panel, 15/67 had positive mRNA expression levels that overlapped with positive protein levels, 37/67 had some overlap between protein and corresponding mRNA expression and 10/61 did not overlap in either islet and spleen populations (visualized by FeaturePlot and DotPlot). Markers not assessed did not have RNA level equivalents, such as CD45RA. For example, consistent expression patterns of CD3, CD127 and CD14 were observed at both the protein and mRNA levels. In contrast CD8, CD4, CD56 and CD138 were not highly expressed at the mRNA level but could be readily detected at the protein level. (**Figure 8A**). Importantly, protein level data within the CITE-seq dataset increased the resolution and accuracy of our annotations. For example, T cell populations can be more clearly delineated by distinct surface level CD4 and CD8 expression and likewise, cytotoxic T cell and NK cell populations can be delineated with clear expression of surface CD56.

We also compared mRNA to protein expression on islet- and spleen-derived T cells to determine whether protein level quantification may have been affected by digestion (**Figures 8B, C**). T cells from the merged spleen and islet Seurat object were re-clustered (**Figure 8B**), and T-cell-associated gene and protein expression were compared between digested (islet), and undigested (spleen) tissue. While all 67 CITE-seq proteins were assessed, we focused on analysis of CD4, CD8A, CD25, PD1, CD27 and CCR6 (**Figure 8C**), since flow cytometry studies identified these markers as being highly susceptible to digestion (**Figure 3**). Minimal differences in protein expression between the two measurement methods were observed.

## 4 Discussion

In this study we examined strategies to optimize CITE-seq of resident immune cells by testing epitope susceptibility to an enzymatic digestion cocktail commonly used to isolate islets, titrating antibodies for CITE-seq by flow cytometry, and comparing CITE-seq data generated with 67 oligo-conjugated antibodies in parallel samples of spleen and islet immune cells. This work revealed epitopes that are highly susceptible to enzymatic digestion, but that were still detectable on cells within the islet capsule. Parallel analysis of flow cytometry and CITE-seq-detected protein expression revealed good data concordance and enabled identification of islet resident immune cells that would have been difficult to detect on the basis of mRNA expression alone.

Digestion of the exocrine pancreas in collagenase is a well-established method for human islet isolation in preparation for transplantation or for laboratory studies of islets, beta cells and/or islet-resident immune cells (27, 36). To determine whether this process could affect cell surface proteins on immune cells, we performed flow cytometry with PBMCs experimentally exposed to enzymes commonly used in the preparation of islets. These data showed that depending on the antibody clone, the ability to detect CD4, CD8a, CD25, CD27, CCR6, and PD-1 can be affected by enzymatic digestion. In accordance with our findings, CD4, CD8a, CD25, and CD27 have also been observed by other groups to be sensitive to digestion (24).

To further assess the impact of collagenase on cell surface marker expression by CITE-seq, we compared protein and mRNA expression in digested islets and mechanically dissociated spleen, focusing on the proteins found to be most cleavage-susceptible by flow cytometry. Contrary to flow cytometry data, protein expression of CD8a and PD-1 was higher in islets (digested) than in spleen (undigested). These findings suggest that while these epitopes are sensitive to enzymatic digestion, CITE-seq has sufficient sensitivity to detect expression. Similar expression of CD4 in islets and spleen at the protein and mRNA was observed, suggesting that digestion did not negatively affect the ability to detect this marker by CITE-seq. We did not detect CD25 or CD27 protein expression on immune cells from human islets but could observe immune cells expressing these markers in spleen. This may be a result of collagenase-induced cleavage, but as reported by other groups (15) it is more likely that CD25^+^ cells are not present in islet-resident immune cells from people without T1D, as it was also not detected by flow cytometry or at the mRNA level. Further, *FOXP3* mRNA (expressed by CD25^hi^ Tregs) was not detected. We noted low *CD27* gene expression in both tissues, making it unclear if the difference in CD27 expression between islets and spleen are a result of collagenase-induced cleavage or reflective of biological differences in immune cells from these distinct tissues. Finally, we observed no CCR6 protein or mRNA expression in either islets or spleen, so while this marker is sensitive to digestion, it was likely not expressed by immune cells within tissues studied within this donor.

Surface protein cleavage by collagenase can be impacted by the length of digestion time and concentration of the enzyme. In human islet isolation protocols, collagenase is typically delivered *via* the common bile duct for dispersal throughout the pancreas (26, 36). Exposure of islet-resident immune cells to collagenase during this process varies among donors, collagenase lots (36), and islet preparations. We theorize that the epitopes of immune cells within islets may have been shielded by cells that comprise the islet mantle, minimizing the exposure of immune cells to digestion enzymes and mitigating any potential proteolytic effect. Thus, our assessment of the impact of collagenase on PBMCs may not enable direct comparison to islet-resident immune cell cleavage after digestion. Nonetheless, identifying the epitopes most susceptible to cleavage is still important to determine whether differences in cell surface expression might be biological or related to the islet digestion protocol. Our study highlights the utility of parallel sequencing of control tissues (e.g., splenocytes) that do not require enzymatic digestion to create single cell suspensions, whenever possible.

Titrating oligo-conjugated antibodies for multimodal single-cell analysis is important for improving sensitivity, lowering background signal, and reducing sequencing costs (23). In our study, we did 3-point serial dilutions with the middle concentration being suggested by the manufacturer (BioLegend) using PE-conjugated versions of the same clones used for CITE-seq. We noted that the manufacturer’s recommended antibody staining concentration, which had been optimized on circulating immune cells in blood, was only optimal for 15 of 67 antibodies, suggesting antibody optimization may have to be tailored for tissue studies. By using splenocytes rather than PBMCs for optimizations, we were better able to select a concentration which had the optimal balance between positive signal and low background. This observation is of note, as unlike antibody titrations for flow cytometry, with CITE-seq it is important to not use saturating amounts of antibody, as this may result in sequencing of unbound, aggregated antibodies and reduced overall sequencing depth (23).

Due to the high cost of sequencing and the rarity of human islet donors, we performed our antibody titrations using flow cytometry analysis of human splenocytes, rather than with human islets and sequencing. While this enhanced our ability to select optimal antibody dilution for most markers, it may also have led to sub-optimal selection of CITE-seq antibody concentrations for islet studies. We noted that while helpful for many epitopes, the use of PE-conjugated antibodies as a proxy for CITE-seq oligo-tagged antibodies was imperfect. This could in part be due to the variance in conjugation stoichiometry that is often not consistent between varying formats and batches of fluorescent antibodies (37). It may also be due to differences in sensitivity of ADT sequencing as compared to fluorescent antibody detection (38). While flow based titration methods are used widely as an economical and relatively quick method of antibody optimizations for CITEseq studies, this may lead to using sub-optimal CITE-seq antibody concentrations. Despite this, in cases where there was a background signal, we were able to perform manual modifications of signal expression to visualize the differential expression, leading us to conclude that oligo-conjugated antibody titration by flow cytometry is for the most part a helpful technique to obtain concentrations needed for CITE-seq but the source of immune cells can influence selection of appropriate concentration.

CITE-seq allows for optimal annotation of cell populations and identification of rare cells that cannot be identified by RNA sequencing alone. We found that protein expression data from 67 surface markers increased our ability to annotate myeloid cells, T cells, NK cells and other ILCs, as well as mast cells in human islets. As ILCs are present in low abundance in islets, and can have very similar profiles at the RNA level to T cell populations, including expression of CD3 at the transcriptional level (39, 40), we enriched NK cells and helper ILCs by flow cytometry-sorting prior to sequencing. CITE-seq enabled clear differentiation of helper ILCs from T cells by positive expression of CD127 without high surface CD4 and CD8 protein expression. There were discrepancies between mRNA and protein expression for ILC markers including *IL7R* and CD127, *KLRD1* and CD94, and CD56 and *NCAM1*. The latter is particularly relevant, as CD56 is expressed by several ILCs – NK cells, key among – but also natural killer T cells and activated T cells. The poor capture of *NCAM1* in mRNA data illustrates the benefit of CITE-seq versus single cell RNA sequencing to identify CD56-expressing cells (41).

In summary, we found that although digestion of immune cells with islet isolation enzymes has the potential to significantly affect cell surface expression of several epitopes, the structure of islets might mitigate any proteolytic effects on resident immune cells. With the use of accurately titrated antibodies, CITE-seq adds a valuable layer of information to single-cell RNA sequencing in the characterization of islet-resident immune cell subsets. This work sets the stage for a more comprehensive investigation of how these cells change in health and disease.

## Data availability statement

The original contributions presented in the study are publicly available. This data can be found here: Gene Expression Omnibus, GSE224767.

## Ethics statement

The studies involving human participants were reviewed and approved by University of British Columbia Clinical Research Ethics Board (B22-0075 and H18-02553), Canadian Blood Services, and the University Health Network Research Ethics Board (17-6229 and 20-5206). The patients/participants provided their written informed consent to participate in this study.

## Author contributions

SJC, MB, and MM contributed equally to this work and share first authorship. CV, ML, and SQC contributed equally to this work and share senior authorship. KR, JM, and SI contributed to experimental design and analysis. SJC, MB, MM, CV, ML, and SQC wrote the manuscript, which all authors reviewed and edited. All authors contributed to the article and approved the submitted version.

## References

[1] Burrack AL Martinov T Fife BT . T Cell-mediated beta cell destruction: Autoimmunity and alloimmunity in the context of type 1 diabetes. Front Endocrinol (2017) 8:343. doi: 10.3389/fendo.2017.00343 PMC5723426 29259578

[2] Dalmas E Lehmann FM Dror E Wueest S Thienel C Borsigova M . Interleukin-33-Activated islet-resident innate lymphoid cells promote insulin secretion through myeloid cell retinoic acid production. Immunity (2017) 47(5):928–942.e7. doi: 10.1016/j.immuni.2017.10.015 29166590

[3] Molofsky AB Nussbaum JC Liang HE Van Dyken SJ Cheng LE Mohapatra A . Innate lymphoid type 2 cells sustain visceral adipose tissue eosinophils and alternatively activated macrophages. J Exp Med (2013) 210(3):535–49. doi: 10.1084/jem.20121964 PMC3600903 23420878

[4] Xiao X Gittes GK . Concise review: New insights into the role of macrophages in β-cell proliferation. Stem Cells Transl Med (2015) 4(6):655–8. doi: 10.5966/sctm.2014-0248 PMC4449096 25900729

[5] Zhou L He X Cai P Li T Peng R Dang J . Induced regulatory T cells suppress Tc1 cells through TGF-β signaling to ameliorate STZ-induced type 1 diabetes mellitus. Cell Mol Immunol (2021) 698–710. doi: 10.1038/s41423-020-00623-2 PMC8027661 33446887

[6] Budd MA Monajemi M Colpitts SJ Crome SQ Verchere CB Levings MK . Interactions between islets and regulatory immune cells in health and type 1 diabetes. Diabetologia (2021) 64(11):2378–88. doi: 10.1007/s00125-021-05565-6 34550422

[7] Nackiewicz D Dan M Speck M Chow SZ Chen YC Pospisilik JA . Islet macrophages shift to a reparative state following pancreatic beta-cell death and are a major source of islet insulin-like growth factor-1. iScience (2020) 23(1):100775. doi: 10.1016/j.isci.2019.100775 31962237 PMC6971395

[8] Denroche HC Miard S Sallé-Lefort S Picard F Verchere CB . T Cells accumulate in non-diabetic islets during ageing. Immun Ageing. (2021) 18(1):8. doi: 10.1186/s12979-021-00221-4 33622333 PMC7901217

[9] Zinselmeyer BH Vomund AN Saunders BT Johnson MW Carrero JA Unanue ER . The resident macrophages in murine pancreatic islets are constantly probing their local environment, capturing beta cell granules and blood particles. Diabetologia (2018) 61(6):1374–83. doi: 10.1007/s00125-018-4592-4 PMC5938291 29589072

[10] Brissova M Aamodt K Brahmachary P Prasad N Hong JY Dai C . Islet microenvironment, modulated by vascular endothelial growth factor-a signaling, promotes β cell regeneration. Cell Metab (2014) 19(3):498–511. doi: 10.1016/j.cmet.2014.02.001 24561261 PMC4012856

[11] Han JM Wu D Denroche HC Yao Y Verchere CB Levings MK . IL-33 reverses an obesity-induced deficit in visceral adipose tissue ST2+ T regulatory cells and ameliorates adipose tissue inflammation and insulin resistance. J Immunol Baltim Md 1950 (2015) 194(10):4777–83. doi: 10.4049/jimmunol.1500020 25870243

[12] Winer DA Winer S Shen L Wadia PP Yantha J Paltser G . B cells promote insulin resistance through modulation of T cells and production of pathogenic IgG antibodies. Nat Med (2011) 17(5):610–7. doi: 10.1038/nm.2353 PMC3270885 21499269

[13] Grange C Létourneau J Forget MA Godin-Ethier J Martin J Liberman M . Phenotypic characterization and functional analysis of human tumor immune infiltration after mechanical and enzymatic disaggregation. J Immunol Methods (2011) 372(1):119–26. doi: 10.1016/j.jim.2011.07.002 21782822

[14] Quatromoni JG Singhal S Bhojnagarwala P Hancock WW Albelda SM Eruslanov E . An optimized disaggregation method for human lung tumors that preserves the phenotype and function of the immune cells. J Leukoc Biol (2015) 97(1):201–9. doi: 10.1189/jlb.5TA0814-373 PMC4771617 25359999

[15] Radenkovic M Uvebrant K Skog O Sarmiento L Avartsson J Storm P . Characterization of resident lymphocytes in human pancreatic islets. Clin Exp Immunol (2017) 187(3):418–27. doi: 10.1111/cei.12892 PMC5290249 27783386

[16] Waise S Parker R Rose-Zerilli MJJ Layfield DM Wood O West J . An optimised tissue disaggregation and data processing pipeline for characterising fibroblast phenotypes using single-cell RNA sequencing. Sci Rep (2019) 9(1):9580. doi: 10.1038/s41598-019-45842-4 31270426 PMC6610623

[17] de Vos P Smink AM Paredes G Lakey JRT Kuipers J Giepmans BNG . Enzymes for pancreatic islet isolation impact chemokine-production and polarization of insulin-producing β-cells with reduced functional survival of immunoisolated rat islet-allografts as a consequence. PloS One (2016) 11(1):e0147992. doi: 10.1371/journal.pone.0147992 26824526 PMC4732769

[18] Stoeckius M Zheng S Houck-Loomis B Hao S Yeung BZ Mauck WM . Cell hashing with barcoded antibodies enables multiplexing and doublet detection for single cell genomics. Genome Biol (2018) 19(1):224. doi: 10.1186/s13059-018-1603-1 30567574 PMC6300015

[19] Stoeckius M Hafemeister C Stephenson W Houck-Loomis B Chattopadhyay PK Swerdlow H . Simultaneous epitope and transcriptome measurement in single cells. Nat Methods (2017) 14(9):865–8. doi: 10.1038/nmeth.4380 PMC5669064 28759029

[20] Scheyltjens I Van Hove H De Vlaminck K Kancheva D Bastos J Vara-Pérez M . Single-cell RNA and protein profiling of immune cells from the mouse brain and its border tissues. Nat Protoc (2022) 17:1–35. doi: 10.1038/s41596-022-00716-4 35931780

[21] Hao Y Hao S Andersen-Nissen E Mauck WM Zheng S Butler A . Integrated analysis of multimodal single-cell data. Cell (2021) 184(13):3573–3587.e29. doi: 10.1016/j.cell.2021.04.048 34062119 PMC8238499

[22] Mazzurana L Czarnewski P Jonsson V Wigge L Ringnér M Williams TC . Tissue-specific transcriptional imprinting and heterogeneity in human innate lymphoid cells revealed by full-length single-cell RNA-sequencing. Cell Res (2021) 31(5):554–68. doi: 10.1038/s41422-020-00445-x PMC8089104 33420427

[23] Buus TB Herrera A Ivanova E Mimitou E Cheng A Herati RS . Improving oligo-conjugated antibody signal in multimodal single-cell analysis. eLife (2021) 10:e61973. doi: 10.7554/eLife.61973 33861199 PMC8051954

[24] Autengruber A Gereke M Hansen G Hennig C Bruder D . Impact of enzymatic tissue disintegration on the level of surface molecule expression and immune cell function. Eur J Microbiol Immunol (2012) 2(2):112–20. doi: 10.1556/EuJMI.2.2012.2.3 PMC3956959 24672679

[25] Corbin KL West HL Brodsky S Whitticar NB Koch WJ Nunemaker CS . A practical guide to rodent islet isolation and assessment revisited. Biol Proced Online. (2021) 23(1):7. doi: 10.1186/s12575-021-00143-x 33641671 PMC7919091

[26] Lyon J Manning Fox JE Spigelman AF Kim R Smith N O’Gorman D . Research-focused isolation of human islets from donors with and without diabetes at the Alberta diabetes institute IsletCore. Endocrinology (2016) 157(2):560–9. doi: 10.1210/en.2015-1562 26653569

[27] Rheinheimer J Ziegelmann PK Carlessi R Reck LR Bauer AC Leitão CB . Different digestion enzymes used for human pancreatic islet isolation: A mixed treatment comparison (MTC) meta-analysis. Islets (2014) 6(4):e977118. doi: 10.4161/19382014.2014.977118 25437379 PMC4588164

[28] Ivison S Malek M Garcia RV Broady R Halpin A Richaud M . A standardized immune phenotyping and automated data analysis platform for multicenter biomarker studies. JCI Insight (2018) 3(23):121867. doi: 10.1172/jci.insight.121867 30518691 PMC6328091

[29] Bossuyt X Marti GE Fleisher TA . Comparative analysis of whole blood lysis methods for flow cytometry. Cytometry (1997) 30(3):124–33. doi: 10.1002/(SICI)1097-0320(19970615)30:3<124::AID-CYTO3>3.0.CO;2-L 9222098

[30] Lex A Gehlenborg N Strobelt H Vuillemot R Pfister H . UpSet: Visualization of intersecting sets. IEEE Trans Vis Comput Graph. (2014) 20(12):1983–92. doi: 10.1109/TVCG.2014.2346248 PMC4720993 26356912

[31] Protocol - TotalSeq™-b or -c with 10x feature barcoding technology. Available at: https://www.biolegend.com/en-us/protocols/totalseq-b-or-c-with-10xfeature-barcoding-technology.

[32] Hafemeister C Satija R . Normalization and variance stabilization of single-cell RNA-seq data using regularized negative binomial regression. Genome Biol (2019) 20:296. doi: 10.1186/s13059-019-1874-1 31870423 PMC6927181

[33] Stuart T Butler A Hoffman P Hafemeister C Papalexi E Mauck WM . Comprehensive integration of single-cell data. Cell (2019) 177(7):1888–1902.e21. doi: 10.1016/j.cell.2019.05.031 31178118 PMC6687398

[34] Becht E McInnes L Healy J Dutertre CA Kwok IWH Ng LG . Dimensionality reduction for visualizing single-cell data using UMAP. Nat Biotechnol (2018) 38–44. doi: 10.1038/nbt.4314 30531897

[35] Korsunsky I Millard N Fan J Slowikowski K Zhang F Wei K . Fast, sensitive and accurate integration of single-cell data with harmony. Nat Methods (2019) 16(12):1289–96. doi: 10.1038/s41592-019-0619-0 PMC6884693 31740819

[36] Kin T Zhai X Murdoch TB Salam A Shapiro AMJ Lakey JRT . Enhancing the success of human islet isolation through optimization and characterization of pancreas dissociation enzyme. Am J Transplant Off J Am Soc Transplant Am Soc Transpl Surg (2007) 7(5):1233–41. doi: 10.1111/j.1600-6143.2007.01760.x 17359501

[37] Ivell R Teerds K Hoffman GE . Proper application of antibodies for immunohistochemical detection: Antibody crimes and how to prevent them. Endocrinology (2014) 155(3):676–87. doi: 10.1210/en.2013-1971 PMC3929726 24428532

[38] Stoeckius M Hafemeister C Stephenson W Houck-Loomis B Chattopadhyay PK Swerdlow H . Large-Scale simultaneous measurement of epitopes and transcriptomes in single cells. Nat Methods (2017) 14(9):865–8. doi: 10.1038/nmeth.4380 PMC5669064 28759029

[39] Björklund ÅK Forkel M Picelli S Konya V Theorell J Friberg D . The heterogeneity of human CD127 + innate lymphoid cells revealed by single-cell RNA sequencing. Nat Immunol (2016) 17(4):451–60. doi: 10.1038/ni.3368 26878113

[40] Suffiotti M Carmona SJ Jandus C Gfeller D . Identification of innate lymphoid cells in single-cell RNA-seq data. Immunogenetics (2017) 69(7):439–50. doi: 10.1007/s00251-017-1002-x 28534222

[41] Lawlor N Nehar-Belaid D Grassmann JDS Stoeckius M Smibert P Stitzel ML . Single cell analysis of blood mononuclear cells stimulated through either LPS or anti-CD3 and anti-CD28. Front Immunol (2021) 12:636720. doi: 10.3389/fimmu.2021.636720 33815388 PMC8010670

