# Supplementary material for: Strategies for optimizing CITE-seq for human islets and other tissues
Source: Front Immunol. 2023 Mar 1;14:1107582. doi: 10.3389/fimmu.2023.1107582 (PMC10014726; doi:10.3389/fimmu.2023.1107582)
Supplement: Supplementary file 1 [file DataSheet_1.pdf]

Supplemental Data

Strategies for optimizing CITE-seq for human islets and other tissues

Sarah J. Colpitts[*] [a,b], Matthew A. Budd[*] [c,d], Mahdis Monajemi[*] [c,d], Kyle T. Reid[a,b], Julia M. Murphy[a,b], Sabine M. Ivison [c,d], , C. Bruce Verchere[†] [c,d,e], Megan K. Levings[†] [c,d,f] Sarah Q. Crome[†,a,b]

[*] Department of Immunology, Temerty Faculty of Medicine, University of Toronto
[*] Toronto General Hospital Research Institute, Ajmera Transplant Centre, University Health Network
[*] Department of Surgery, University of British Columbia, Vancouver BC, Canada
[*] BC Children's Hospital Research Institute, Vancouver BC, Canada
[*] Department of Pathology & Laboratory Medicine, University of British Columbia, Vancouver BC, Canada and Centre for Molecular Medicine and Therapeutics
[*] School of Biomedical Engineering, University of British Columbia, Vancouver BC, Canada

Corresponding authors:
Megan K. Levings
mlevings@bcchr.ca

Sarah Q. Crome
sarah.crome@utoronto.ca

C. Bruce Verchere
bverchere@bcchr.ca

---

[*] These authors contributed equally to this work and share first authorship
[†] These authors contributed equally to this work and share senior authorship

Supplemental Figures

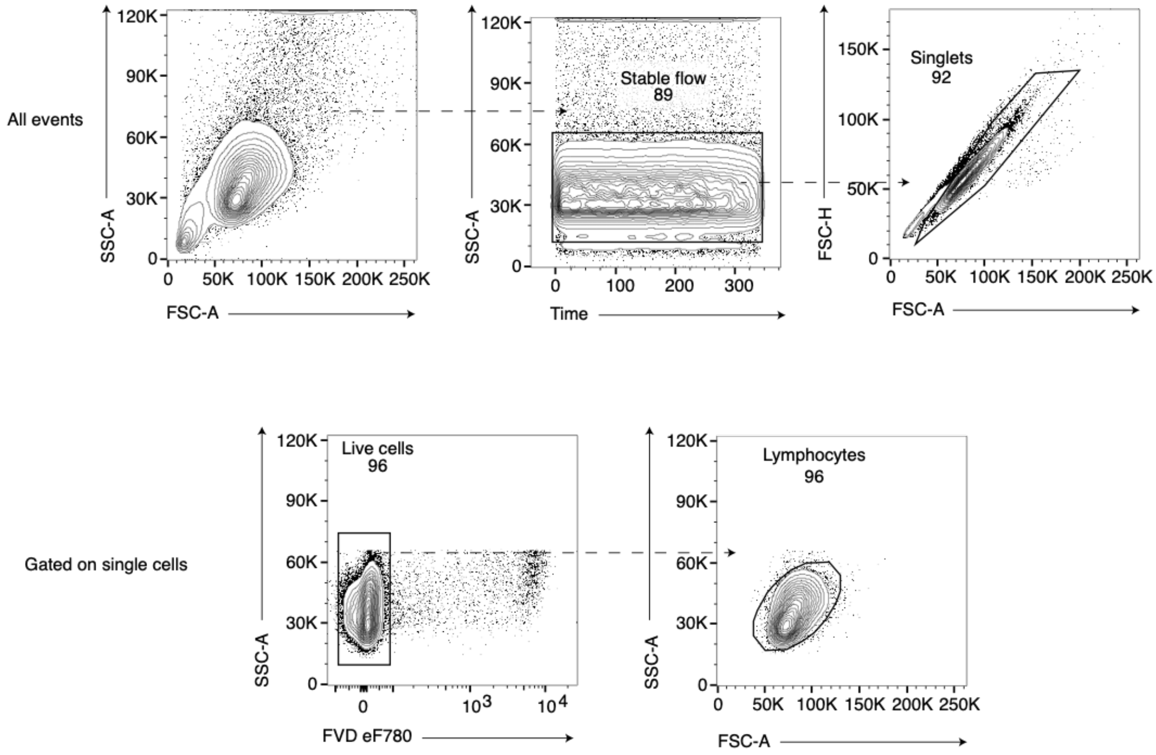

Supplemental Figure S1. Strategies for gating parent cell populations and comparing the staining of flow cytometry antibodies following treatment with a digestive enzyme panel.

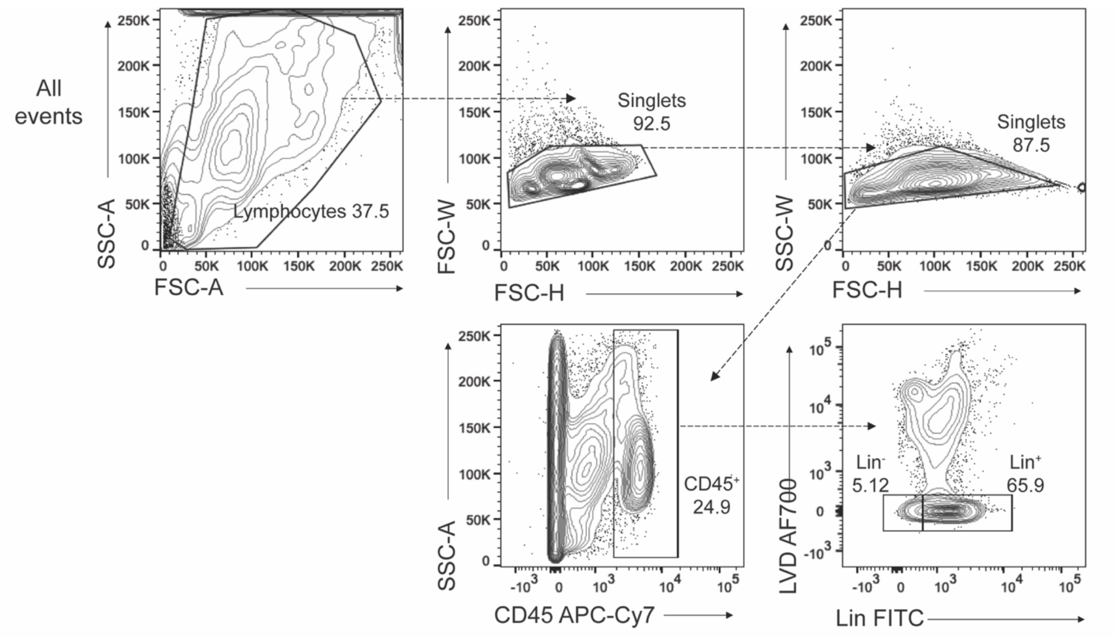

Supplemental Figure S2. Gating strategy to sort Lin⁻ innate lymphoid cells (ILCs).

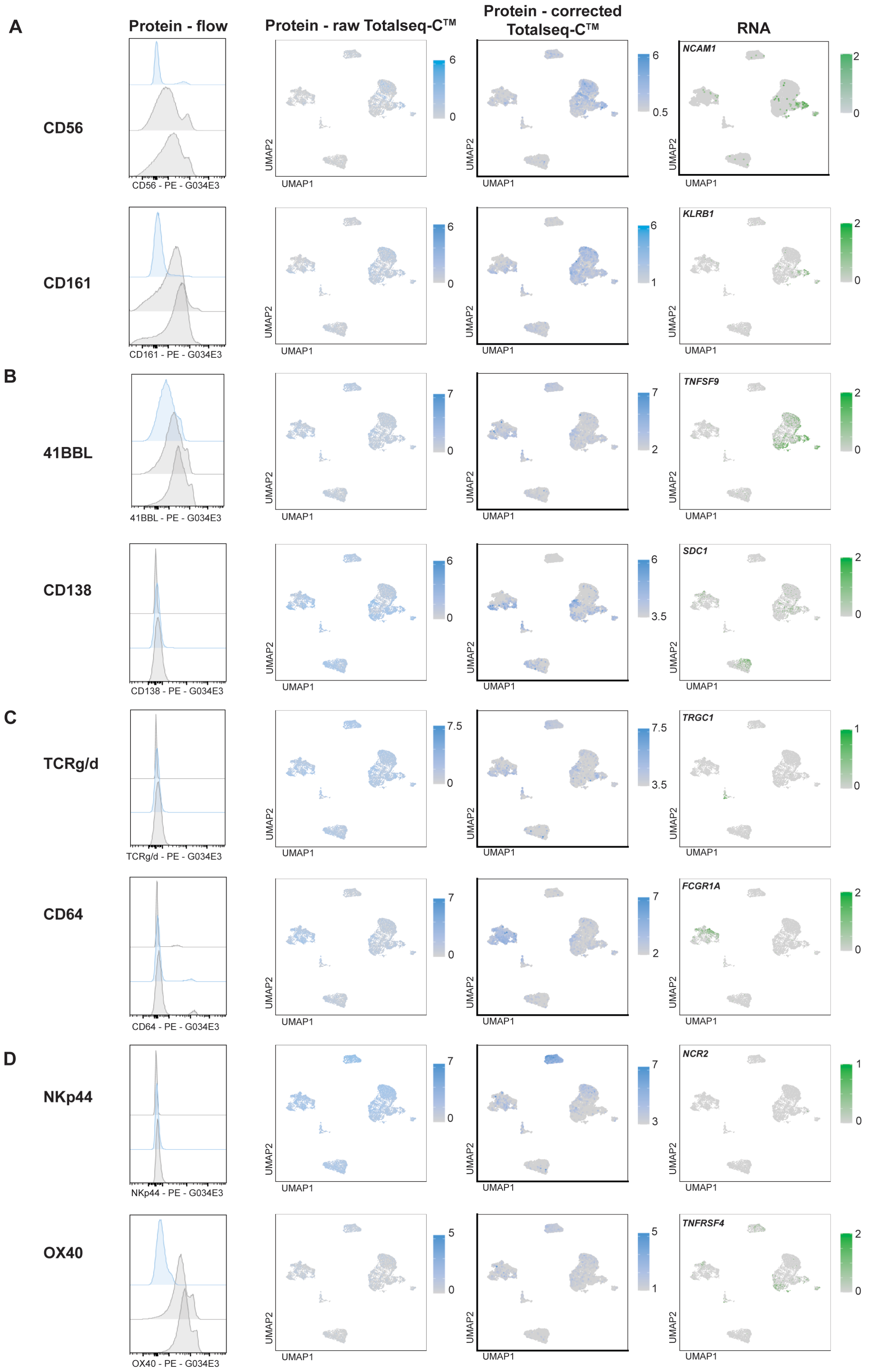
A
Protein - flow
Protein - raw Totalseq-C™
Protein - corrected Totalseq-C™
RNA
CD56
CD56 - PE - G034E3
NCAM1
CD161
CD161 - PE - G034E3
KLRB1
B
41BBL
41BBL - PE - G034E3
TNFSF9
CD138
CD138 - PE - G034E3
SDC1
C
TCRg/d
TCRg/d - PE - G034E3
TRGC1
CD64
CD64 - PE - G034E3
FCGR1A
D
NKp44
NKp44 - PE - G034E3
NCR2
OX40
OX40 - PE - G034E3
TNFRSF4
UMAP1
UMAP2

Supplemental Figure S3. Adjusting background signal of oligo-conjugated antibodies allows for clear protein signal by CITE-seq even if concentrations based on flow cytometric calculations are suboptimal. Splenocytes were stained with 3 concentrations of the indicated PE-labelled mAbs and analyzed by flow cytometry. The histogram representing the concentration used for CITE-seq is indicated in blue. Using combined spleen and islet data, the corresponding UMAPs were generated from ADT (antibody-derived tag, blue) and RNA data (green). Corrected UMAPs were generated by adjusting the minimum expression to reduce background signal. (A) Examples of markers where CITE-seq antibody concentration was ideal based on signal to noise ratio and resulted in little background expression by ADT. (B) Examples of markers where concentration selected was ideal based on signal to noise ratio but which resulted in background ADT expression. (C) Examples of markers where CITE-seq antibody concentration was higher than ideal based on high signal to noise ratio and high background expression by ADT was observed. (D) Examples of markers for which CITE-seq antibody concentration was lower than ideal based on low signal to noise ratio but which gave high background ADT signal.

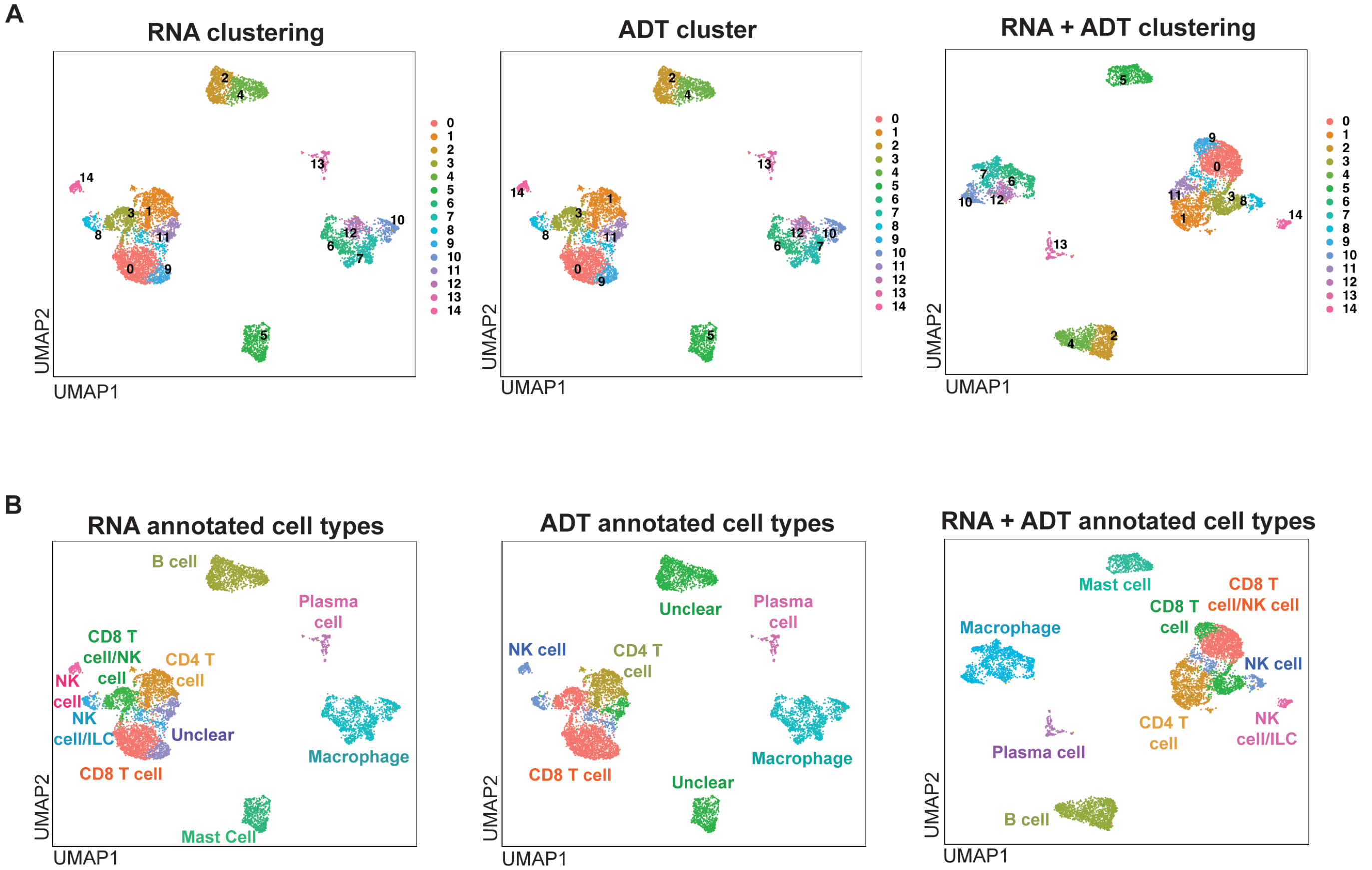

Supplemental Figure S4. Clustering with RNA only or ADT (antibody-derived tags) only does not allow accurate cell type annotations. (A) UMAP of the clustering of immune cells derived from the merged spleen/islets with only RNA data, only ADT data and both RNA and ADT data at 0.75 resolution. (B) Annotation of cell types based on RNA only clustering, ADT only clustering and combined RNA and ADT clustering.

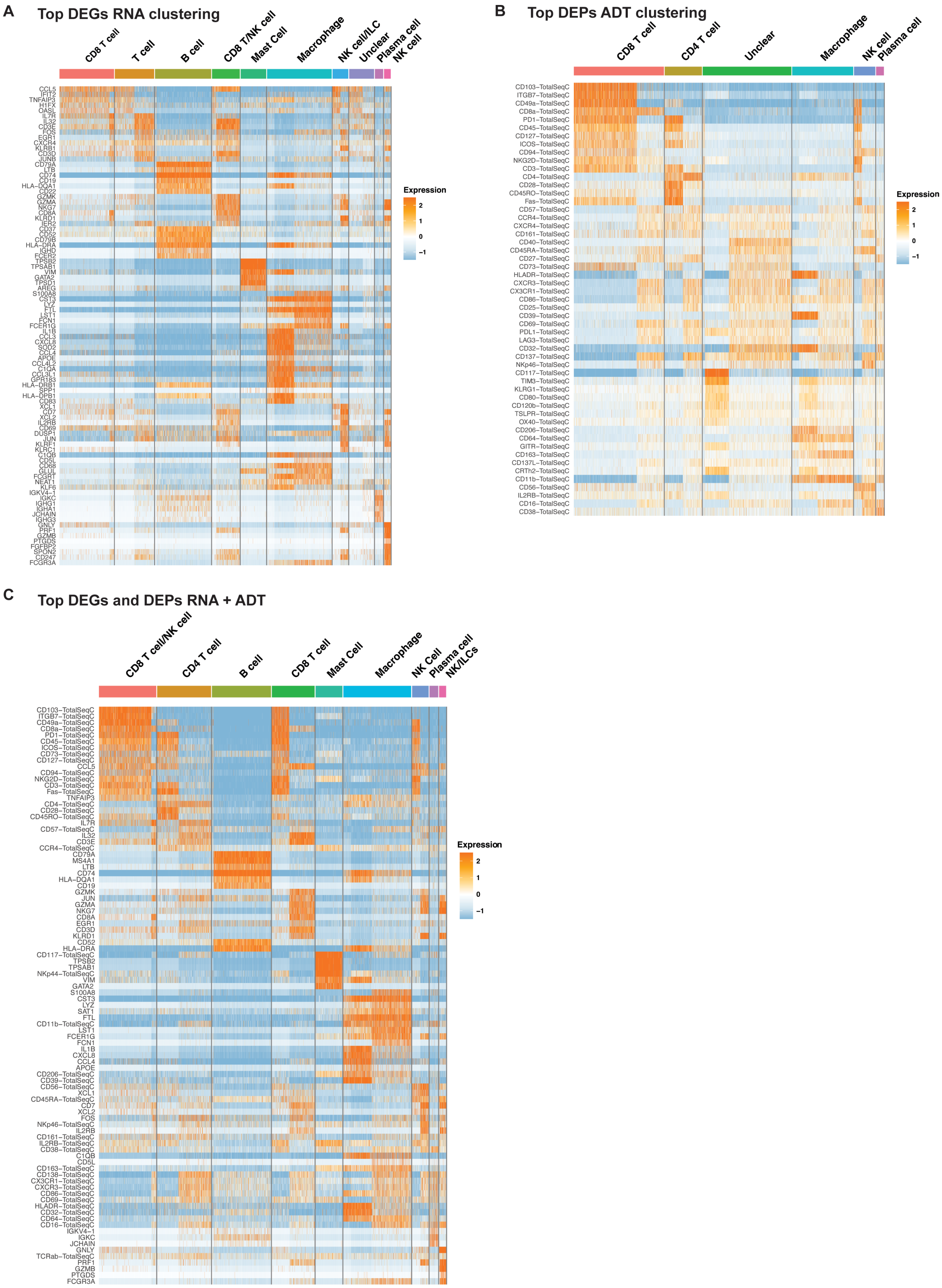
A
Top DEGs RNA clustering
CD8 T cell
T cell
B cell
CD8 T/NK cell
Mast Cell
Macrophage
NK cell/ILC
Unclear
Plasma cell
NK cell
CCL5
IFIT2
TNFAIP3
H1FX
OASL
IL7R
IL32
CD3E
FOS
EGR1
CXCR4
KLRB1
CD3D
JUNB
CD79A
LTB
CD74
CD19
HLA−DQA1
CD22
GZMK
GZMA
NKG7
CD8A
KLRD1
IER2
CD37
CD52
CD79B
HLA−DRA
IGHD
FCER2
TPSB2
TPSAB1
VIM
GATA2
TPSD1
AREG
S100A8
CST3
LYZ
FTL
LST1
FCN1
FCER1G
IL1B
CCL3
CXCL8
SOD2
CCL4
APOE
CCL4L2
C1QA
CCL3L1
GPR183
HLA−DRB1
SPP1
HLA−DPB1
CD83
XCL1
CD7
XCL2
IL2RB
CD69
DUSP1
JUN
KLRF1
KLRC1
C1QB
CD5L
CD68
GLUL
FCGRT
NEAT1
KLF6
IGKV4−1
IGKC
IGHG1
IGHA1
JCHAIN
IGHG3
GNLY
PRF1
GZMB
PTGDS
FGFBP2
SPON2
CD247
FCGR3A
Expression
2
1
0
−1
B
Top DEPs ADT clustering
CD8 T cell
CD4 T cell
Unclear
Macrophage
NK cell
Plasma cell
CD103−TotalSeqC
ITGB7−TotalSeqC
CD49a−TotalSeqC
CD8a−TotalSeqC
PD1−TotalSeqC
CD45−TotalSeqC
CD127−TotalSeqC
ICOS−TotalSeqC
CD94−TotalSeqC
NKG2D−TotalSeqC
CD3−TotalSeqC
CD4−TotalSeqC
CD28−TotalSeqC
CD45RO−TotalSeqC
Fas−TotalSeqC
CD57−TotalSeqC
CCR4−TotalSeqC
CXCR4−TotalSeqC
CD161−TotalSeqC
CD40−TotalSeqC
CD45RA−TotalSeqC
CD27−TotalSeqC
CD73−TotalSeqC
HLADR−TotalSeqC
CXCR3−TotalSeqC
CX3CR1−TotalSeqC
CD86−TotalSeqC
CD25−TotalSeqC
CD39−TotalSeqC
CD69−TotalSeqC
PDL1−TotalSeqC
LAG3−TotalSeqC
CD32−TotalSeqC
CD137−TotalSeqC
NKp46−TotalSeqC
CD117−TotalSeqC
TIM3−TotalSeqC
KLRG1−TotalSeqC
CD80−TotalSeqC
CD120b−TotalSeqC
TSLPR−TotalSeqC
OX40−TotalSeqC
CD206−TotalSeqC
CD64−TotalSeqC
GITR−TotalSeqC
CD163−TotalSeqC
CD137L−TotalSeqC
CRTh2−TotalSeqC
CD11b−TotalSeqC
CD56−TotalSeqC
IL2RB−TotalSeqC
CD16−TotalSeqC
CD38−TotalSeqC
Expression
2
1
0
−1
C
Top DEGs and DEPs RNA + ADT
CD8 T cell/NK cell
CD4 T cell
B cell
CD8 T cell
Mast Cell
Macrophage
NK Cell
Plasma cell
NK/ILCs
CD103−TotalSeqC
ITGB7−TotalSeqC
CD49a−TotalSeqC
CD8a−TotalSeqC
PD1−TotalSeqC
CD45−TotalSeqC
ICOS−TotalSeqC
CD73−TotalSeqC
CD127−TotalSeqC
CCL5
CD94−TotalSeqC
NKG2D−TotalSeqC
CD3−TotalSeqC
Fas−TotalSeqC
TNFAIP3
CD4−TotalSeqC
CD28−TotalSeqC
CD45RO−TotalSeqC
IL7R
CD57−TotalSeqC
IL32
CD3E
CCR4−TotalSeqC
CD79A
MS4A1
LTB
CD74
HLA−DQA1
CD19
GZMK
JUN
GZMA
NKG7
CD8A
EGR1
CD3D
KLRD1
CD52
HLA−DRA
CD117−TotalSeqC
TPSB2
TPSAB1
NKp44−TotalSeqC
VIM
GATA2
S100A8
CST3
LYZ
SAT1
FTL
CD11b−TotalSeqC
LST1
FCER1G
FCN1
IL1B
CXCL8
CCL4
APOE
CD206−TotalSeqC
CD39−TotalSeqC
CD56−TotalSeqC
XCL1
CD45RA−TotalSeqC
CD7
XCL2
FOS
NKp46−TotalSeqC
IL2RB
CD161−TotalSeqC
IL2RB−TotalSeqC
CD38−TotalSeqC
C1QB
CD5L
CD163−TotalSeqC
CD138−TotalSeqC
CX3CR1−TotalSeqC
CXCR3−TotalSeqC
CD86−TotalSeqC
CD69−TotalSeqC
HLADR−TotalSeqC
CD32−TotalSeqC
CD64−TotalSeqC
CD16−TotalSeqC
IGKV4−1
IGKC
JCHAIN
GNLY
TCRab−TotalSeqC
PRF1
GZMB
PTGDS
FCGR3A
Expression
2
1
0
−1

Supplemental Figure S5. Combined RNA and ADT clustering more accurately delineates cell types than RNA or ADT alone. Heatmap representation of top differentially expressed genes (DEGs) and differentially expressed proteins (DEPs) from each cluster categorized by the cluster's annotated cell type using only RNA data (A), only ADT data (B), or combined RNA + ADT data (C).

Supplemental Tables

Supplemental Table S1. Donor information per experiment.

Assay performed	Tissue source	Age	Sex	Cause of Death	Diabetes
CITE-seq	Islets	40	Male	NDD - Neurological	None
Flow cytometry	Islets	70	Male	MAID - Medical Assistance in Dying	None
Flow cytometry	Islets	32	Male	NDD - Neurological	None
Flow cytometry	Islets	57	Female	NDD - Neurological	None
Flow cytometry	Islets	41	Male	NDD - Neurological	None
Flow cytometry titrations	Spleen	55	Female	Haemorrhage	None
Flow cytometry digestion studies	PBMC	50	Male	N/A	None
Flow cytometry digestion studies	PBMC	30	Male	N/A	None
Flow cytometry digestion studies	PBMC	33	Female	N/A	None
Flow cytometry digestion studies	PBMC	45	Male	N/A	None

Supplemental Table S2. Flow cytometry antibodies tested.

Antigen	Clone	Fluorophore	Manufacturer
CD3	UCHT1	BB515	BD
		BUV395	BD
		BUV737	BD
CD4	13B8.2	APC	Beckman Coulter
	OKT4	APC	Invitrogen
		BV421	BioLegend
		BV510	BioLegend
		BV570	BioLegend
		BV711	BioLegend
	RPA-T4	AF700	BD
		FITC	Invitrogen
		V500	BD
	SFCI12T4D11	PE-Cy7	Beckman Coulter
	SK3	BUV395	BD
CD8a	B9.11	PE-Cy5.5	Beckman Coulter
	HIT8a	FITC	Invitrogen
	OKT8	APC	Invitrogen
	RPA-T8	BUV563	BD
	SK1	BV785	BioLegend
		PE-Cy7	BD
		PerCP-Cy5.5	BD
CD11b	ICRF44	PE-CF594	BD
CD11c	B-Ly6	APC	BD
CD14	M5E2	BV785	BD
CD16	3G8	BUV395	BD
		BUV805	BD
CD19	SJ25C1	BV711	BD
		PE-Cy7	BD

CD25	2A3	BUV395	BD
		BV711	BD
	4E3	PE	BioLegend
	B1.49.9	PE	BioLegend
	BC96	BV421	BioLegend
		BV650	BioLegend
	M-A251	APC	BD
		FITC	BD
		PE-Cy7	BD
CD27	1A4CD27	ECD	Beckman Coulter
	O323	AF700	Invitrogen
		BV605	BioLegend
		BV711	BioLegend
CD28	CD28.2	APC	BioLegend
CD32	FUN-2	PE	BioLegend
CD38	HIT2	BV421	BioLegend
CD39	A1	FITC	BioLegend
CD40	5C3	PE	BioLegend
		PE-Cy7	BD
CD45	2D1	FITC	BioLegend
	HI30	V500	BD
CD45RA	HI100	PE-Cy7	Invitrogen
CD45RO	UCHL1	BUV395	BD
CD49a	TS2/7	PE	BioLegend
CD49b	AK-7	AF647	BD
	P1E6-C5	PE	BioLegend
CD49d	9F10	PE	BioLegend

CD56	5.1H11	PE	BioLegend
CD57-Rec	QA17A04	PE	BioLegend
CD64	10.1	BUV737	BD
CD69	FN50	BV785	BioLegend
		FITC	Invitrogen
		PE-Cy7	BD
CD80	2D10	PE	BioLegend
	L307.4	BUV496	BD
CD86	IT2.2	PE-Cy7	Invitrogen
CD94	DX22	PE	BioLegend
CD95	DX2	BV605	BioLegend
		FITC	BD
CD107a	H4A3	PE	BioLegend
CD117	104D2	BV421	BD
		PE-Cy7	BioLegend
CD120b	3G7A02	PE	BioLegend
CD122	TU27	PE	BioLegend
CD127	A019D5	APC	BioLegend
		FITC	BioLegend
	HIL-7RM21	BV605	BD
CD134	Ber-ACT35 (ACT35)	PE	BioLegend
CD137	4B4-1	APC	BioLegend
		PE-Cy7	BioLegend

CD137L	5F4	PE	BioLegend
CD138	MI15	PerCP-Cy5.5	BioLegend
CD152	BNI3	BV786	BD
CD161	HP-3G10	BV421	BioLegend
		PE-Cy7	Invitrogen
CD163	GHI/61	BV421	BD
CD183	G025H7	AF488	BioLegend
		BV421	BioLegend
CD184	12G5	BV711	BD
CD194	1G1	PE-Cy7	BD
	L291H4	APC	BioLegend
		BV605	BioLegend
		PE-Dazzle594	BioLegend
CD196	11A9	PE	BioLegend
	G034E3	APC	BioLegend
		BV605	BioLegend
		BV785	BioLegend
	R6H1	APC	Invitrogen
CD197	G043H7	PerCP-Cy5.5	BioLegend
CD206	15-2	PE	BioLegend
CD223	11C3C65	PE	BioLegend
	3DS223H	PE-Cy7	Invitrogen
	Polyclonal	PE	R&D Systems
	REA351	APC	Miltenyi
	T47-530	PE	BD
CD274	29E.2A3	BV785	BioLegend
		PE	BioLegend

CD278	C398.4A	BV510	BioLegend
CD279	eBioJ105	APC-eF780	Invitrogen
		PerCP-eF710	Invitrogen
	EH12.1	BUV737	BD
	EH12.2H7	BV421	BD
	PD1.3	APC	Beckman Coulter
CD294	BM16	BV421	BD
		PE-CF594	BD
CD314	1D11	PE	BioLegend
CD335	9E2	BV711	BioLegend
CD336	P44-8	BB515	BD
CD357	108-17	PE	BioLegend
GARP	7B11	BUV737	BD
HLA-DR	L243	BV510	BioLegend
KLRG1	2F1/KLRG1	BV711	BioLegend
TCRVα24-Jα18	6B11	PE	BioLegend
TCRα/β	IP26	BV480	BD
TCRγ/δ	11F2	BB660-P2	BD
		PE-Cy7	BD
	B1	APC-Fire750	BioLegend
		PE	BioLegend
TIGIT	A15153G	BV421	BioLegend
TSLPR	1D3	PE	BioLegend

Supplemental Table S3. TotalSeq™-C antibodies used for CITE-seq staining.

Antibody	ug per 100 ul/1 million cells
0047 anti-human CD56	0.05
0149 anti-human CD161	0.125
0061 anti-human CD117 (c-kit)	0.25
0083 anti-human CD16	0.025
0089 anti-human TIGIT (VSTM3)	0.125
0101 anti-human CD335 (NKp46)	0.05
0102 anti-human CD294 (CRTH2)	1.25
0390 anti-human CD127 (IL-7Rα)	0.05
0143 anti-human CD196 (CCR6)	0.0125
0165 anti-human CD314 (NKG2D)	0.53125
0802 anti-human CD336 (NKp44)	1.25
0867 anti-human CD94	0.025
0153 anti-human KLRG1 (MAFA)	0.1
0140 anti-human CD183 (CXCR3)	0.05
0139 anti-human TCR γ/δ	0.425
0048 anti-human CD45	0.01
0584 anti-human TCR Vα24-Jα18 (iNKT cell)	0.5
0224 anti-human TCR α/β	0.015
0080 anti-human CD8a	0.025
0034 anti-human CD3	0.0125
0072 anti-human CD4	0.025
0055 anti-human CD138 (Syndecan-1)	0.425
0081 anti-human CD14	0.025
0205 anti-human CD206 (MMR)	0.25
0159 anti-human HLA-DR	0.106
0063 anti-human CD45RA	0.03125
0087 anti-human CD45RO	0.125
0085 anti-human CD25	0.025
0152 anti-human CD223 (LAG-3)	0.125
0179 anti-human CX3CR1	0.0625
0387 anti-human TSLPR (TSLP-R)	0.5
0371 anti-human CD49b	0.025
0389 anti-human CD38	0.05
0168 anti-human CD57 Recombinant	0.025
0575 anti-human CD49a antibody	0.025
0171 anti-human/mouse/rat CD278 (ICOS)	0.0625
0360 anti-human CD357 (GITR)	0.5

0176 anti-human CD39	0.0125
0146 anti-human CD69	0.025
0088 anti-human CD279 (PD-1) Antibody	0.125
0151 anti-human CD152 (CTLA-4)	0.25
0155 anti-human CD107a (LAMP-1)	0.0625
0156 anti-human CD95 (Fas)	0.125
0158 anti-human CD134 (OX40)	0.125
0022 anti-human CD137L (4-1BB Ligand)	0.5
0031 anti-human CD40	0.025
0355 anti-human CD137 (4-1BB)	1.0625
0071 anti-human CD194 (CCR4)	0.10625
0154 anti-human CD27	0.0425
0386 anti-human CD28	0.03125
0901 anti-human GARP (LRRC32)	0.125
0246 anti-human CD122 (IL-2Rβ)	0.0625
0366 anti-human CD184 (CXCR4)	0.5
0576 anti-human CD49d	0.0125
0007 anti-human CD274 (B7-H1, PD-L1)	0.0625
1024 anti-human CD120b	0.255
0005 anti-human CD80	0.625
0142 anti-human CD32/ Fcg RII	0.10625
0161 anti-human CD11b	0.2
0162 anti-human CD64 (FCGR1A)	0.10625
0006 anti-human CD86	0.0425
0358 anti-human CD163	0.53125
0148 anti-human CD197 (CCR7)	0.5
0577 anti-human CD73	0.5
0214 anti-human/mouse integrin β7	0.5
0169 anti-human CD366 (Tim-3)	1
0145 anti-human CD103 (Integrin αE)	0.5
